# VLM-Based Mesh Segmentation Framework for DDH Robotic Ultrasound

**DOI:** 10.3390/bioengineering13101213

**Published:** 2026-10-17

**Authors:** Yuanbo Wang, Jianwei Cui

**Affiliations:** Institute of Instrument Science and Engineering, Southeast University, Nanjing 210096, China; 220233566@seu.edu.cn

**Keywords:** structured vision–language model, multi-view semantic segmentation, 3D mesh cleanup, infant phantom

## Abstract

For robotic ultrasound targeting developmental dysplasia of the hip (DDH), reconstructed infant body-surface meshes should preserve the target surface while excluding bed, support, and background geometry. This study proposes a structured vision–language model (VLM)-based multi-view segmentation method for infant phantom mesh cleanup. Text-guided 2D masks provide foreground and background evidence; mask-quality statistics and structured VLM assessments generate view-specific foreground-support weights that undergo deterministic risk suppression before target/non-target classification of 3D vertices. Hard voting and Rule-weighted voting were baselines under identical projection and post-processing settings. We evaluated one physical infant phantom across 150 separate acquisition–reconstruction trials under controlled conditions, each comprising 40 voting views and 10 held-out evaluation views, yielding 1500 manually annotated reference masks. Structured VLM voting achieved a Balanced score (intersection over union minus the 2-pixel tolerant leakage rate) of 0.804 ± 0.085, versus 0.801 ± 0.084 and 0.793 ± 0.086 for Rule-weighted and Hard voting. This leakage rate was 0.087 ± 0.043, versus 0.089 ± 0.042 and 0.092 ± 0.043; mean Coverage remained similar (0.992–0.993). The Balanced-score gain and leakage-rate reduction relative to Rule-weighted voting remained significant after Holm correction (adjusted *p* = 0.029 and *p* = 0.033, respectively). Validation using real-infant data, 3D reference standards, and closed-loop robotic scanning is still required.

## 1. Introduction

Developmental dysplasia of the hip (DDH) encompasses abnormalities ranging from mild hip instability to complete femoral-head dislocation; delayed detection can lead to early osteoarthritis, limping, and lasting functional impairment [1]. In infants, clinical examination and risk assessment are commonly combined with imaging, and ultrasonography is particularly important before femoral-head ossification [2]. Recent artificial-intelligence studies have addressed wireless handheld ultrasound screening, segmentation, angle measurement, diagnosis, automated radiographic measurements, and real-time standard-plane detection [3,4,5,6], and have extended into primary-care and selective-screening workflows [7,8]. These advances improve the analysis and quality control of 2D DDH images but do not directly address the body-surface geometry required before autonomous scanning.

Robot-assisted ultrasound places additional demands on geometric preparation because the reachable region, initial probe pose, scan boundaries, and safety constraints must be established before contact. Body-surface geometry is therefore an important input for path planning and contact control. The RobUSt system uses 3D point clouds for path planning and navigation together with impedance control [9]. Automated trajectory generation from 3D point clouds has also been investigated for autonomous ultrasound scanning [10]. Other studies have investigated task-space compliance and six-dimensional force regulation [11] and anatomy-guided scan-path planning from body-surface point clouds [12]. Reviews identify autonomous scanning, path planning, contact control, and image guidance as central components of robotic ultrasound [13]. Representative systems have also integrated automated scanning with musculoskeletal segmentation and 3D reconstruction [14] or robotic Doppler imaging of lower-extremity arteries [15]. In such pipelines, residual bed, support, or background geometry can introduce spurious surface points and normals, shift the estimated scanning region, or generate invalid path points, potentially reducing planning accuracy and distorting the geometric constraints used for collision avoidance and safe probe approach. This literature primarily addresses scanning, control, image acquisition, and reconstruction; the preparation of a clean infant body-surface mesh as an upstream geometric input remains less explicitly studied.

Text-guided detection and promptable segmentation offer a practical way to obtain 2D body-surface evidence with limited annotations. The Segment Anything Model (SAM) generates masks from visual prompts such as points or bounding boxes [16], whereas Grounding DINO localizes open-set objects from text descriptions [17]. Medical adaptations include MedSAM across imaging modalities [18], MedCLIP-SAM for text-prompted medical image segmentation [19], and AutoSAMUS for automatically prompted ultrasound image segmentation [20]. These models support candidate body-surface mask generation, but occlusion, reflections, visually similar backgrounds, fragmentation, and boundary drift can make individual masks unreliable. When projected across views, such errors may accumulate as incorrect 3D geometry.

Mapping 2D semantics into 3D representations commonly relies on cross-view projection or feature fusion. Classical multi-view geometry addresses inconsistent observations through robust model fitting, as exemplified by RANSAC [21], and through pixelwise view selection in unstructured multi-view stereo [22]; these geometric checks complement semantic mask-quality assessment. Three-dimensional Gaussian Splatting (3DGS) represents scenes with optimized Gaussian primitives for real-time radiance-field rendering [23], while Two-dimensional Gaussian Splatting (2DGS) improves multi-view geometric reconstruction and supports surface-mesh extraction [24]. Building on Gaussian representations, LangSplat embeds image–text features in 3D Gaussian representations [25]; Gaussian Grouping uses 2D SAM masks for 3D scene grouping [26]; and Segment Anything in 3D propagates SAM masks across views in a neural radiance field [27]. OpenMask3D and Open3DIS use multi-view image features or 2D mask guidance for open-vocabulary 3D instance segmentation [28,29], while Segment Any 3D Gaussians transfers 2D segmentation into Gaussian representations [30]. These semantic-fusion studies establish that 2D semantic evidence can constrain 3D representations, but they mainly target scene querying, grouping, or 3D segmentation rather than cleaning an existing surface mesh while controlling the influence of unreliable views.

Variation in view reliability further motivates the use of vision–language models (VLMs). VividMed demonstrates medical visual grounding through semantic masks and instance-level bounding boxes [31], whereas MLeVLM organizes medical visual information across recognition, detail, diagnosis, knowledge, and reasoning [32]. In general vision–language modeling, CLIP established transferable image–text representations [33], and Qwen-VL supports image understanding, visual grounding, and text recognition [34]. A Qwen2-VL model adapted using biomedical video data has also demonstrated domain-specific learning on medical and surgical video question-answering benchmarks [35]. Although these studies do not directly evaluate multi-view mask quality, their capabilities support structured assessment of mask completeness, background leakage, and preservation of DDH-relevant body regions.

**Figure 1:**
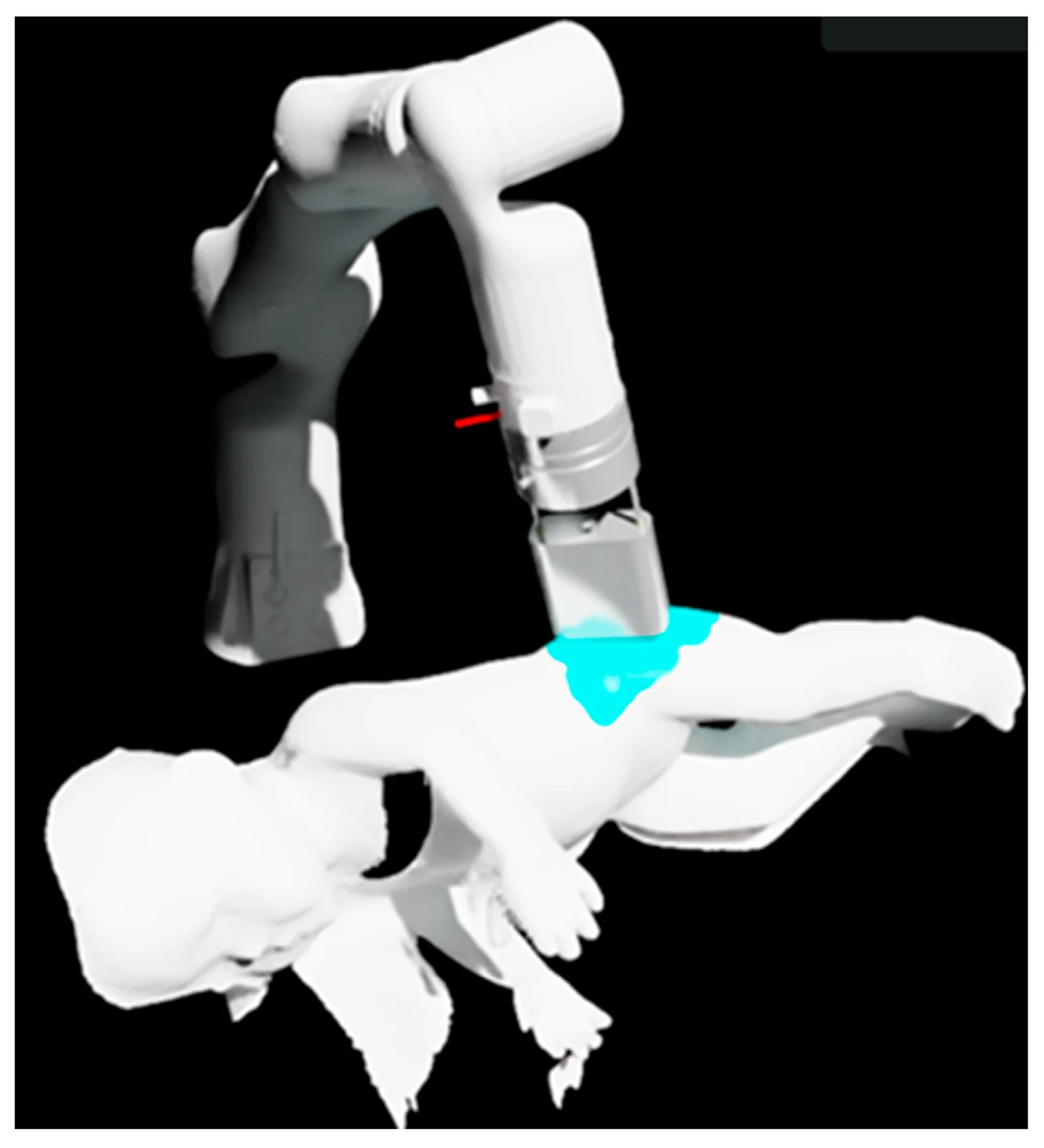
Downstream robotic-ultrasound simulation scene in NVIDIA Isaac Sim using the cleaned infant-phantom surface mesh and a robot-mounted simulated ultrasound probe model. The cyan area denotes the intended target body-surface region; the red segment indicates the axis direction of the robot end-effector.

In the present setting, upstream multi-view reconstruction of an infant phantom produces an initial mesh that may include the bed surface, supports, background cloth, pads, and thin attached structures. We therefore define mesh cleanup as binary target/non-target classification of 3D vertices using multi-view 2D segmentation evidence, followed by removal of non-target geometry and connected-component post-processing, rather than general multi-class 3D semantic segmentation. The primary methodological contribution of this study is a structured VLM-guided view-weighting strategy that, unlike equal-weight voting or weighting based only on predefined mask statistics, converts structured assessments of mask completeness, background leakage, and preservation of DDH-relevant body regions into explicit foreground-support weights, followed by deterministic risk suppression within a unified 3D vertex-voting framework. As a supporting implementation and evaluation contribution, we integrate text-guided 2D mask generation, view-quality assessment, mesh cleanup, and held-out reprojection evaluation into an end-to-end pipeline and compare Hard voting, Rule-weighted voting, and Structured VLM voting across 150 separate acquisition–reconstruction trials of one physical infant phantom. The cleaned infant-phantom mesh was also imported into NVIDIA Isaac Sim to construct a downstream robotic-ultrasound simulation scene incorporating a robot-mounted simulated ultrasound probe model for subsequent studies of probe positioning, scanning-region definition, and path planning (Figure 1); this scene was not part of the quantitative method comparison. Accordingly, the present study does not evaluate DDH diagnosis, ultrasound-image interpretation, probe–surface contact control, or closed-loop robotic scanning.

## 2. Materials and Methods

To obtain infant body surface geometry suitable for initial probe positioning, scanning-range constraints, and path planning in robotic ultrasound, this study developed a 3D mesh cleanup framework jointly driven by 2D semantic evidence and view-quality assessment to address residual bed surfaces, supports, and background structures commonly present in multi-view reconstructed meshes. The framework formulates mesh cleanup as a 3D vertex-retention decision under multi-view observations. Body surface masks generated from individual views provide foreground and background evidence, while mask-statistics rules and a structured vision–language model (VLM) assess the reliability of evidence from different views. The target body surface mesh is subsequently obtained through weighted voting and connected-component post-processing. This design aims to suppress non-target geometry such as bed surfaces and supports while reducing the excessive removal of key body surface regions caused by low-quality views or local mask errors.

**Figure 2:**
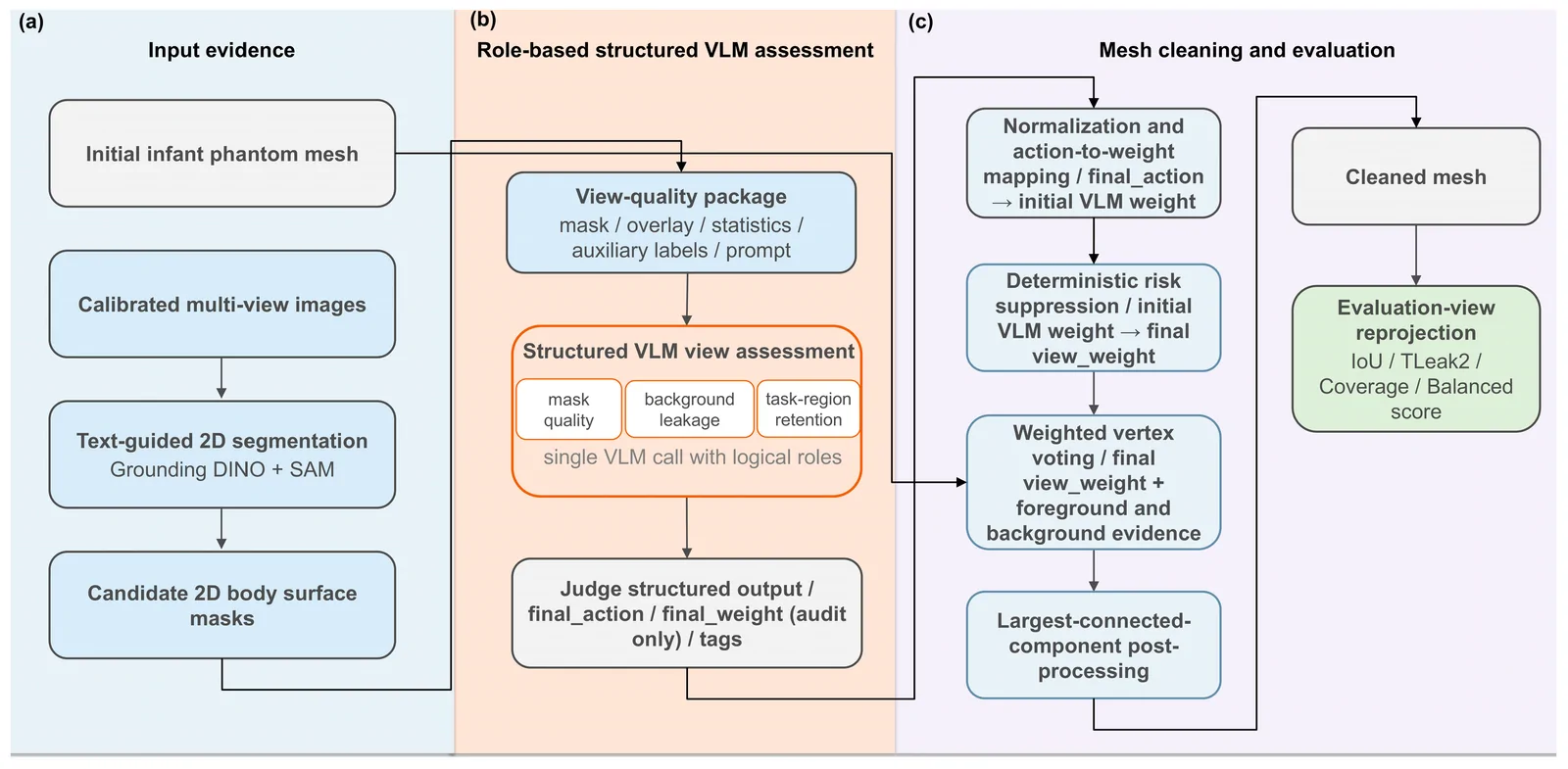
Workflow of the proposed structured VLM-guided mesh-cleanup method: (**a**) input evidence construction; (**b**) structured view-quality assessment; and (**c**) view weighting, mesh cleanup, and held-out reprojection evaluation.

The overall workflow of the proposed method is illustrated in Figure 2 and comprises three main stages: input evidence construction, structured view-quality assessment, and mesh cleanup and evaluation. The initial infant phantom mesh and calibrated multi-view images serve as parallel inputs. The calibrated images are processed using Grounding DINO and SAM to generate candidate 2D body surface masks, which are combined with mask overlays, quality statistics, auxiliary labels, and structured prompts to construct view-quality information packages. Within a single VLM call, logical roles for mask quality, background leakage, and task-region retention assess the reliability of the semantic evidence in each voting view, and the Judge Agent synthesizes these assessments into a final disposition action and other structured outputs. After normalization and fallback handling, the final disposition action is mapped to an initial VLM weight. Deterministic risk suppression then constrains this initial weight to obtain the final view weight. The foreground and background evidence from the voting views is subsequently projected onto the initial mesh, and the cleaned mesh is generated through weighted vertex voting and largest-connected-component post-processing. Finally, the cleaned mesh is reprojected onto evaluation views that were not involved in semantic cleanup and compared with manually annotated reference masks using IoU, the 2-pixel tolerant leakage rate, Coverage, and the Balanced score.

### 2.1. Study Subject, Data Sources, and Task Scope

This study addressed the extraction and cleanup of infant phantom surface meshes for robotic ultrasound preprocessing. A commercially available infant phantom (model KF; purchased from Fujun Industrial Co., Ltd., Shanghai, China) was used in this study. The phantom measured 52 cm in height and was made entirely of vinyl. The experimental data were obtained from 150 separate multi-view acquisition–reconstruction trials conducted using this infant phantom. Each trial included a newly acquired image sequence, camera parameters, and an initial 3D mesh generated by the upstream 2DGS reconstruction pipeline [24]. The mesh generated by 2DGS was used as the initial geometric input, and the study focused on cleaning this mesh by integrating 2D semantic evidence with view-quality assessment.

Each acquisition–reconstruction trial comprised 99–155 camera views, with a median of 121. The raw meshes contained 222,509–2,281,001 vertices and 426,109–4,418,718 faces. The 150 trials covered variations in phantom pose, background or support-surface color, support structure, illumination condition, camera trajectory, and acquisition session. All trials were performed using the same infant phantom and therefore primarily reflect engineering repeatability under different controlled acquisition conditions rather than inter-subject variability. Because data acquisition from real infants and repeated annotation are subject to safety and ethical constraints, an infant phantom was used as the engineering validation platform. The generalizability of the findings should therefore be limited to this phantom and the acquisition conditions covered in this study.

### 2.2. Inclusion and Exclusion Criteria and View Partitioning

The inclusion criteria were as follows: each acquisition–reconstruction trial was required to contain complete multi-view images, camera parameters, and a readable raw mesh; image references had to correspond one-to-one with the camera records; all three primary comparison methods had to generate readable cleaned meshes; and the corresponding evaluation views had to contain manually annotated reference masks that passed quality control. The exclusion criteria included missing images, camera parameters, or raw meshes; manually annotated reference masks that failed quality control; or cleaned meshes from any primary comparison method that were missing, empty, or unreadable.

Using a prespecified deterministic equal-interval rule, 40 voting views and 10 evaluation views were selected from the camera sequence of each trial. The voting views were used for text-guided 2D mask generation, view-quality assessment, and 3D vertex voting, whereas the evaluation views did not participate in semantic cleanup and were used only for the final reprojection evaluation. It should be noted that these 10 evaluation views were still involved in the upstream 2DGS reconstruction but not in semantic cleanup. They were therefore defined as evaluation views specifically for the semantic cleanup stage.

To assess sensitivity to the number of voting views, deterministic nested equal-interval subsets were constructed from the ordered 40-view set for $K\in \left\{10,20,30,40\right\}$. The (K=10) subset used positions 1, 5, …, 37; (K=20) used the odd-numbered positions from 1 to 39; (K=30) combined the (K=20) subset with positions 2, 6, …, 38; and (K=40) used all prespecified voting views. Each configuration was evaluated across the same 150 trials and 10 evaluation views. To distinguish the effect of view count from that of threshold scaling, the analysis used both the fixed primary retention threshold (T=4) and a proportional threshold $T_{K}=max(1,round(\frac{4K}{40}))$. For computational feasibility, all K-sensitivity configurations were evaluated using the same batch OpenCV 4.13.0 cv2.fillPoly rasterizer. Comparisons among K values were therefore interpreted only within this common rasterization backend and were not made directly against the absolute values from the PIL-based primary evaluation.

Sensitivity to the number of evaluation views was examined for $M\in \left\{2,4,6,8,10\right\}$ by enumerating all $\left(\begin{array}{c} 10\\ M \end{array}\right)$ subsets of the 10 prespecified evaluation-view positions. For each subset, the same positional indices were applied across all 150 trials, and the existing per-view reprojection metrics were reaggregated without rerunning mesh projection. Both view-count analyses were descriptive and did not alter the prespecified primary configuration comprising 40 voting views (K=40) and 10 evaluation views (M=10).

### 2.3. 2D Body Surface Mask Generation and View-Quality Statistics

For each voting view, Grounding DINO was first used to localize candidate regions of the infant phantom surface based on the text prompt “the smooth skin of the baby doll.” The detected bounding boxes were then provided to SAM to generate binary foreground masks. Grounding DINO used the Swin Transformer-Tiny (Swin-T) OGC configuration with a bounding-box score threshold of 0.35, while SAM used the Vision Transformer-Base (ViT-B) architecture. If multiple candidate masks were present in the same view, they were merged to form the final foreground mask for that view. If no valid candidate region was detected, the view was recorded as having an empty mask.

Mask-quality statistics were subsequently calculated for each voting view, including the foreground-pixel ratio, candidate bounding-box area ratio, number of connected components, proportion of foreground pixels in the largest connected component, and Laplacian blur score. Rule-based quality labels were generated from these statistics. A foreground-pixel ratio below 0.05 was labeled as a low foreground ratio, whereas a ratio above 0.65 was labeled as a high foreground ratio. A mask with more than eight connected components was labeled as having multiple connected regions, whereas a largest-connected-component foreground ratio below 0.95 was labeled as mask fragmentation. A blur score below 30.0 indicated a low-clarity view. These statistics were recorded for quality assessment. The rule-derived labels and associated statistics were used to assign view weights in the Rule-weighted voting method, whereas the full set of statistics and labels was included in the view-quality information package for Structured VLM assessment.

To assess whether the findings depended on these nominal values, a prespecified one-factor-at-a-time sensitivity analysis evaluated the nominal configuration and 15 alternative settings. The low-foreground-ratio threshold was varied to 0.10, 0.15, and 0.20; the high-foreground-ratio threshold to 0.30 and 0.33; the connected-component-count threshold to 2, 4, 12, and 16; the largest-connected-component-ratio threshold to 0.45 and 1.00; and the blur-score threshold to 10.0, 20.0, 45.0, and 60.0. For each alternative setting, only the specified threshold was changed, while the other four thresholds, the original 2D masks and VLM outputs, the 150 separate acquisition–reconstruction trials, the 40 voting views, the 10 evaluation views, the vertex-retention threshold, and all projection, mesh post-processing, and evaluation definitions were held fixed. Threshold classification in this analysis used full-precision statistics recomputed from the original image and mask files rather than rounded display values. All three comparison methods were evaluated under every setting. The analysis was descriptive: the nominal thresholds were not reselected on the basis of the observed results, and no additional significance tests were introduced.

The dataset size, view partitioning, and key parameters for 2D segmentation and 3D voting are summarized in Table 1; the threshold values listed there correspond to the nominal configuration.

### 2.4. Unified Multi-View Semantic Voting Framework and Baseline Methods

To ensure that differences among the three comparison methods arose primarily from the source of the view-specific foreground-support weights, Hard voting, Rule-weighted voting, and Structured VLM voting—the latter described in Section 2.5—all used the same initial mesh, 2D body surface masks, and preselected set of voting views as inputs. They also employed the same vertex-projection implementation, foreground-retention threshold, and mesh post-processing procedure. For each acquisition–reconstruction trial, 40 voting views were selected in advance. If a view lacked a usable mask, had a weight of 0, or could not be projected validly, it produced no valid voting evidence. The principal difference among the three methods was the source of the view-specific foreground-support weights. Hard voting assigned equal weights to all views that actually participated in voting, Rule-weighted voting assigned view weights according to mask-quality statistics, and Structured VLM voting generated view weights through structured quality assessment. This section describes only the unified multi-view semantic voting framework and the two baseline methods: Hard voting and Rule-weighted voting.

#### 2.4.1. Vertex Projection and Definition of Semantic Evidence

Let V denote the vertex set of the initial triangular mesh, and let an arbitrary vertex be denoted by v. Let K denote the number of voting views preselected for each trial; in the main experiment, K=40. For the *i*-th voting view, $a_{i}\left(v\right)\in \{0,1\}$ is defined to indicate whether vertex v can be validly projected in that view, and $m_{i}\left(v\right)\in \{0,1\}$ is defined to indicate whether the projected location of the vertex lies within the binary foreground mask of that view.

To distinguish the preselected voting views from those that actually participated in voting, let $I_{h}$ denote the index set of views that actually participated in Hard voting, and let $I_{w}$ denote the index set of views that actually participated in weighted voting. For the weighted voting methods, only views with usable masks and weights greater than 0 were included in $I_{w}$. Views lacking usable masks, having a weight of 0, or failing to undergo valid projection were excluded from the corresponding set. The view weight was denoted by $w_{i}$ for Rule-weighted voting and by $w_{i}^{final}$ for Structured VLM voting.

Specifically, the code first reads the camera rotation matrix using the camera-to-world convention and transposes it to obtain the world-to-camera rotation matrix. The translation term is then calculated from the camera position in the world coordinate system, and the mesh vertices are transformed into the camera coordinate system. Let the coordinates of a vertex in the camera coordinate system be $\left(x_{c},y_{c},z_{c}\right)$, the camera intrinsic parameters be $f_{x}$ and $f_{y}$, and the image width and height be w and h, respectively. The image center is defined as follows:

(1) $$c_{x}=\frac{w}{2},\;c_{y}=\frac{h}{2}$$

The continuous projection coordinates of the vertex are calculated as follows:

(2) $$u_{img}=\frac{x_{c}f_{x}}{z_{c}}+c_{x},\;v_{img}=\frac{y_{c}f_{y}}{z_{c}}+c_{y}$$

Because $u_{img}$ and $v_{img}$ obtained through perspective projection are generally continuous values, whereas the binary mask uses integer pixel indices, the mask value was sampled using the nearest-neighbor method. Here, $round\left(\cdot \right)$ denotes rounding a continuous projection coordinate to the nearest integer pixel coordinate. A projection was considered valid only when the depth satisfied $z_{c}>0.05$ and the rounded pixel coordinates fell within the image boundaries, i.e.,

(3) $$0\leq round\left(u_{img}\right)<w,\;0\leq round\left(v_{img}\right)<h$$

Only under these conditions was the vertex considered to have a valid projection in the current view, and $a_{i}\left(v\right)=1$; otherwise, $a_{i}\left(v\right)=0$. For a valid projected location, nearest-neighbor sampling was performed using the rounded integer pixel coordinates, and the semantic evidence was defined according to the value of that pixel in the binary foreground mask:

(4) $$m_{i}\left(v\right)=\left\{\begin{array}{ll} 1, & the\;projected\;pixel\;lies\;within\;the\;foreground\;mask,\\ 0, & the\;projected\;pixel\;lies\;outside\;the\;foreground\;mask \end{array}\right.$$

Thus, $a_{i}\left(v\right)m_{i}\left(v\right)$ represents the foreground-support evidence provided by the *i*-th view for vertex v, whereas $a_{i}\left(v\right)\left[1-m_{i}\left(v\right)\right]$ represents the background-opposition evidence provided by that view. Views lacking a valid mask, having a weight of 0, or not actually participating in voting generated no valid evidence for the corresponding vertex.

The main experiment used the direct-projection approximation described above without additional explicit z-buffer occlusion testing, back-face culling, or normal-angle filtering. After vertex selection, all three methods applied the same mesh post-processing procedure. Connected components were first identified based on triangle adjacency, and only the largest connected triangular component in terms of face count was retained. Vertices no longer referenced by any triangle were then removed to obtain the final cleaned mesh.

#### 2.4.2. Hard Voting

Hard voting treats all views that actually participate in voting and have valid projections as equally weighted evidence. Each view contributes one vote to both foreground support and background opposition, without differentiating among views based on mask-quality statistics.

For vertex v, the foreground-support vote count $F\left(v\right)$ is defined as follows:

(5) $$F\left(v\right)=\sum_{i\in I_{h}}a_{i}\left(v\right)m_{i}\left(v\right)$$

The background-opposition vote count $B\left(v\right)$ is defined as follows:

(6) $$B\left(v\right)=\sum_{i{\in I}_{h}}a_{i}\left(v\right)\left[1-m_{i}\left(v\right)\right]$$

Let T denote the foreground-retention threshold, which was set to T=4.0 in this study. The retention decision for vertex v is given by:

(7) $$R_{h}\left(v\right)=1\Leftrightarrow F\left(v\right)\geq T\wedge F\left(v\right)>B\left(v\right)$$

When $F\left(v\right)=B\left(v\right)$, the vertex is not retained because the foreground support must be strictly greater than the background opposition. In other words, a vertex must receive at least four valid foreground votes, and its foreground-support vote count must exceed its background-opposition vote count, before it can enter the subsequent mesh-construction stage.

The same foreground-retention threshold was applied to Hard voting, Rule-weighted voting, and Structured VLM voting. With the voting-view number fixed at K=40, a prespecified sensitivity analysis evaluated integer threshold values from T=2 through T=8 for all three methods. This analysis was distinct from the proportional threshold adjustment $T_{K}$ used in the view-count analysis described in Section 2.2. Across the tested threshold values, the original 2D masks, view-specific weights, 150 separate acquisition–reconstruction trials, 40 voting views, 10 evaluation views, projection procedure, mesh post-processing, PIL-based reprojection evaluation, and metric definitions were held fixed. The nominal value T=4 remained the prespecified setting for the primary analysis; the sensitivity analysis was descriptive and was not used to select an optimal threshold.

Hard voting does not differentiate among views according to mask quality and therefore provides a clear equal-weight baseline for assessing whether the introduction of rule-based quality weights can reduce the influence of low-quality views on erroneous foreground retention.

#### 2.4.3. Rule-Weighted Voting

Rule-weighted voting uses the mask-quality statistics obtained in Section 2.3 to assign a weight $w_{i}$ to each voting view, where $0\leq w_{i}\leq 1$. The statistics recorded in the current implementation include the foreground-pixel ratio, foreground-mask bounding-box area ratio, number of connected components, proportion of foreground pixels in the largest connected component, and Laplacian blur score. The foreground-mask bounding-box area ratio is used only for quality recording and auditing and does not directly participate in the current rule-based weight mapping. The mask-quality states identified from the remaining statistics, their triggering conditions, and the corresponding weights are presented in Table 2.

When multiple quality labels are triggered for the same view, the current code does not multiply, average, or accumulate the corresponding weights; instead, the final weight is determined according to a predefined rule priority. Severe labels have the highest priority, including empty_mask and high_fg_ratio, both of which correspond to a weight of 0.00. These are followed by the fragmentation-related labels many_components and fragmented_mask, which correspond to a weight of 0.50. If none of these conditions is satisfied but a general quality issue is present, the weight is set to 0.70. For example, when many_components and low_blur_score occur simultaneously, the final weight is 0.50 rather than (0.50 × 0.70). These thresholds were determined from typical mask-failure patterns observed in preliminary experiments and were kept fixed throughout all formal experiments. Reprojection results from the evaluation views were not used for parameter tuning.

During 2D mask generation, if Grounding DINO fails to detect a valid candidate bounding box, an empty mask is generated and labeled empty_mask. During 3D voting, views with a weight of 0 are skipped before vertex projection and therefore contribute neither foreground support nor background opposition. For views satisfying $0<w_{i}\leq 1$ and actually participating in voting, the main experiment adopts a conservative voting mode in which foreground support is accumulated according to the view weight, whereas background opposition retains unit strength. The weighted foreground support is defined as follows:

(8) $$F_{w}\left(v\right)=\sum_{i\in I_{w}}w_{i}a_{i}\left(v\right)m_{i}\left(v\right)$$

The background opposition is defined as follows:

(9) $$B_{w}\left(v\right)=\sum_{i{\in I}_{w}}a_{i}\left(v\right)\left[1-m_{i}\left(v\right)\right]$$

The final retention condition is defined as follows:

(10) $$R_{w}\left(v\right)=1\Leftrightarrow F_{w}\left(v\right)\geq T\wedge F_{w}\left(v\right)>B_{w}\left(v\right),\;T=4.0$$

The purpose of this conservative weighting strategy is to reduce the contribution of low-quality views to foreground support for retaining a region as part of the body surface while preserving their opposing evidence against the background, bed surface, and supports. Consequently, low-quality views are less likely to independently cause non-target geometry to be retained by exceeding the foreground threshold, yet they can still contribute to excluding regions classified as background by their masks. Views with a weight of 0 do not belong to the set of views that actually participate in voting and therefore accumulate neither foreground support nor background opposition.

To assess whether the findings depended on the numerical scaling of the view weights, a prespecified sensitivity analysis applied the following power transformation to the nominal final view weights used by Rule-weighted and Structured VLM voting:

(11) $$w_{i}^{\left(\gamma \right)}=(w_{i}{)}^{\gamma },\;w_{i}\in [0,1],\;\gamma \in \{0.50,0.75,1.00,1.25,1.50\}$$

Here, $w_{i}\in \left[0,1\right]$ denotes the nominal final weight of the i-th view, and $w_{i}^{\left(\gamma \right)}$ denotes its transformed value. The setting γ=1.00 reproduces the nominal weight mapping; γ<1.00 moves intermediate weights toward 1, whereas γ>1.00 moves them toward 0, while weights of 0 and 1 remain unchanged. The transformed weight $w_{i}^{\left(\gamma \right)}$ replaced $w_{i}$ only in the foreground-support accumulation, whereas background opposition retained unit strength. Hard voting was not separately re-evaluated because every view actually participating in Hard voting had a weight of 1.00 and was therefore invariant under this transformation. Across the five γ settings, the original 2D masks, rule-derived labels, VLM outputs, 150 separate acquisition–reconstruction trials, 40 voting views, 10 evaluation views, foreground-retention threshold T=4, projection procedure, mesh post-processing, and PIL-based reprojection evaluation were held fixed. The analysis was descriptive; γ=1.00 remained the prespecified nominal setting and the sensitivity results were not used to select a new weight mapping.

Building on the unified voting framework described above, Section 2.5 further introduces the structured VLM-guided method for generating view weights.

### 2.5. Structured VLM-Guided View-Weighted Voting Method

Structured VLM voting shares the unified multi-view vertex-voting framework defined in Section 2.4 with the two preceding methods and differs only in how the view-specific foreground-support weights are generated. The method does not directly segment or modify the 3D mesh, nor does it directly determine whether an individual vertex should be retained. Instead, it evaluates the reliability of the semantic evidence provided by the candidate mask in each 2D voting view and converts the evaluation results into view weights. The final mesh is still generated using the deterministic vertex projection, comparison between foreground support and background opposition, and unified mesh post-processing procedure defined in Section 2.4.

#### 2.5.1. Construction of the View-Quality Information Package

For each voting view, Grounding DINO and SAM first generate a candidate 2D mask, which is overlaid as a yellow region on the original image to produce a mask-overlay image for visual review. The original image, candidate mask, mask-overlay image, quality statistics, rule-based quality labels, and structured prompt are then linked through a unique view identifier to form a single-view quality information package. The mask statistics include the foreground-pixel ratio, candidate bounding-box area ratio, number of connected components, proportion of foreground pixels in the largest connected component, and Laplacian blur score. The rule-based quality labels, rule-derived weight, and corresponding issue tags are also stored in the package as auxiliary information.

The information package organizes the evaluation evidence and supports verification and traceability among the input data, structured assessment results, and final view weights. During the actual VLM call, only the mask-overlay image and structured prompt are provided as inputs. The original image and binary candidate mask are retained solely as traceable resources and are not separately transmitted to the API. The structured prompt incorporates view statistics, task context, role assignments, deterministic decision rules, and a JSON output schema to ensure consistency in the output fields and assessment criteria across different views.

#### 2.5.2. Role-Based Structured VLM Assessment

This study employs a role-based structured assessment within a single VLM call rather than multi-round collaboration among mutually independent agents. Four logical roles are defined in the prompt: the Mask Quality Agent, Background Leakage Agent, Task Region Agent, and Judge Agent. The Mask Quality Agent evaluates whether the yellow candidate mask is complete, continuous, and clear, with particular attention to missing body surface regions, fragmentation, abnormal connected components, and blurring. The Background Leakage Agent assesses whether the mask incorrectly covers supports, pads, cloth, tabletops, or other non-infant regions. The Task Region Agent examines whether DDH-related body surface regions, including the hip, buttocks, abdomen, and thighs, are retained.

The first three roles use the same overlay image, prompt, and set of statistics, with each role producing separate action, weight, tags, and reason fields. The Judge Agent synthesizes the structured assessments of the first three roles and checks the prespecified conservative rules. When key regions are not explicitly retained, severe background leakage is present, or the role-specific assessments conflict with one another, the Judge Agent is not permitted to assign an overly optimistic disposition. When a conflict occurs, the degree of conflict is also recorded, and a more conservative final action is adopted. The inputs, outputs, and verification objectives of each module are presented in Table 3. The complete structured VLM prompt and deterministic decision rules are provided in Supplementary File S1.

#### 2.5.3. Assessment Aggregation and Weight Assignment

The structured JSON output contains four global assessment fields: baby_visible, mask_complete, background_leak, and key_region_kept. Each of the three assessment roles outputs action, weight, tags, and reason, while the Judge Agent additionally outputs conflict_level, final_action, final_weight, final_tags, reflection, and reason.

For the *i*-th view, the disposition action output by the Judge Agent, judge.final_action, is denoted by $c_{i}$. The value of $c_{i}$ is restricted to four categories: keep_high, keep_medium, keep_low, and reject. During post-processing, final_action is read and normalized as the primary decision field. The four action categories are then mapped to 1.00, 0.85, 0.50, and 0.00, respectively, according to the fixed mapping defined in Equation (12), yielding the initial VLM weight. judge.final_weight is retained as an auxiliary field in the raw structured output for result interpretation and runtime auditing, but it does not independently override a valid final_action mapping and does not directly enter the 3D voting equations. Deterministic risk suppression may subsequently reduce the initial weight further, generating the final view weight, view_weight, in the scoring file. The 3D voting stage reads view_weight rather than judge.final_weight. The role-level outputs are used primarily for interpretation, auditing, and conflict tracking and do not independently enter the 3D voting equations.

(12) $$w_{i}^{VLM}=\left\{\begin{array}{ll} 1.00, & c_{i}=keep\text{\_}high,\\ 0.85, & c_{i}=keep\text{\_}medium,\\ 0.50, & c_{i}=keep\text{\_}low,\\ 0.00, & c_{i}=reject. \end{array}\right.$$

If the VLM returns incomplete fields, the code first normalizes the output. Missing role blocks are completed using predefined structures. When Judge fields are missing, a Judge result in the single_agent_fallback format is constructed from the available role outputs. Tags provided as JSON lists or comma-separated strings are parsed into a uniform format; empty tags and the ok tag are removed, and duplicate tags are deduplicated. If an API call or JSON parsing fails, the deterministic failure-handling branch is invoked, and runtime tags such as api_error, qwen_error, data_inspection_failed, or parse_error are appended.

#### 2.5.4. Deterministic Risk Suppression and Mesh Generation

Before 3D voting, deterministic risk suppression is further applied to the initial VLM weight $w_{i}^{VLM}$ to obtain the final voting weight $w_{i}^{final}$. For the *i*-th view, the normalized post-processing tag set comprises the available role-level and Judge-level tags, together with any runtime tags appended during normalization or fallback processing. This step neither invokes the VLM again nor modifies the prompt. Instead, it conservatively downweights high-risk views based on this normalized tag set, the global assessment fields background_leak, key_region_kept, and mask_complete, and the rule-based statistical labels. The current implementation contains no rule that increases a low weight to a higher value; therefore, risk post-processing can only retain, reduce, or reject a view.

To describe this process uniformly, let $\rho_{i}$ denote the single weight cap selected for the i-th view by the first applicable branch of the fixed deterministic priority cascade. The final weight is then defined as follows:

(13) $$w_{i}^{final}=min\left(w_{i}^{VLM},\rho_{i}\right),\;\rho_{i}\in \left\{0.00,0.25,0.50,1.00\right\}$$

When multiple conditions were present for the same view, the implementation did not take the minimum of all tag-associated caps. Instead, the branches were evaluated in the following fixed order: key-region rejection, key-region uncertainty, API fallback, major background leakage, multiple connected components accompanied by leakage, support-related leakage, boundary leakage, preservation of the original action-derived weight, and blur/clean/default handling. The first applicable branch determined $\rho_{i}$. Consequently, the presence of a recorded tag did not necessarily mean that its associated cap controlled the final weight if an earlier branch had already been activated. If no suppressive branch was triggered, $\rho_{i}=1.00$.

Under this strategy, when the key region was classified as no or partial, the final disposition was set to reject and $\rho_{i}=0.00$. When it was classified as uncertain, the disposition was normalized to keep_low and $\rho_{i}=0.50$. A cap of $\rho_{i}=0.00$ was also used for fallback views accompanied by leakage evidence, views with severe background leakage, and views with multiple connected components accompanied by leakage. The support-related branch, including supports, pads, or large-scale background leakage, used $\rho_{i}=0.25$, whereas boundary leakage and fallback views without evident leakage used $\rho_{i}=0.50$. Tags such as major_background_leak, many_components, support_leak, mat_leak, blue_background_leak, large_background_leak, and boundary_leak identified the corresponding risk conditions.

These caps were deterministic engineering risk limits rather than calibrated probabilities or analytically derived constants. The cap $\rho_{i}=0.00$ was reserved for evidence considered unusable for foreground support, whereas $\rho_{i}=0.50$ represented moderate ambiguity. The support-related cap $\rho_{i}=0.25$ provided strong but nonzero suppression because support or large-scale background leakage constitutes clear non-target evidence, while the same mask may still retain usable infant-surface information. Thus, 0.25 lies between complete rejection at 0.00 and the half-weight cap of 0.50 used for less severe boundary-related uncertainty.

To assess sensitivity to this choice, a prespecified offline one-factor-at-a-time analysis varied only the support-related cap as $\rho_{support}\in \left\{0.00,0.25,0.50\right\}$, with $\rho_{support}=0.25$ retained as the nominal setting. All other caps and priority branches, the original 2D masks, rule-derived labels, frozen VLM outputs, 150 separate acquisition–reconstruction trials, frozen view order, K=40 voting views, M=10 held-out evaluation views, foreground-retention threshold T=4, conservative voting rule, projection procedure, mesh post-processing, and PIL-based reprojection evaluation were held fixed. No additional VLM API calls were made. The analysis was descriptive and was not used to select a new cap value.

Finally, $w_{i}^{final}$ is substituted into Equations (8)–(10) in Section 2.4. This weight modulates only the foreground support, whereas background-opposition evidence continues to accumulate with unit strength across the views that actually participate in voting and is not attenuated by the VLM-derived weight. The three methods share the same camera-projection procedure, valid-projection criterion, vertex-retention threshold, and mesh post-processing pipeline, differing only in the source of the view-specific foreground-support weights, as summarized in Table 4.

### 2.6. Manual Reference-Mask Annotation and Mesh Reprojection Evaluation

To establish manual reference standards for the 2D evaluation views, the researcher began with blank masks and manually delineated and verified the visible body surface regions of the infant phantom in each of the 1500 evaluation views before exporting binary manually annotated reference masks. During annotation, the researcher did not inspect the projection results of Hard voting, Rule-weighted voting, or Structured VLM voting, nor were the VLM outputs reviewed. All exported reference masks passed quality checks for file format, dimensions, pixel values, and non-emptiness. These manually annotated reference masks served as image-level 2D reference standards for evaluating the agreement between the projected contours of the cleaned meshes and the visible body surface regions.

After receiving training on the task instructions and boundary-annotation rules, the first annotator completed all 1500 primary reference masks. To assess annotation consistency without modifying the primary reference set, a second trained annotator applied the same predefined annotation protocol to a prespecified subset of 150 held-out views. This subset comprised one view from each of the 150 separate acquisition–reconstruction trials and was balanced across the ten evaluation-view positions, with 15 images selected from each position. The second annotator began with blank masks and did not inspect the first annotator’s masks, the candidate masks, mask-overlay images, quality statistics, or VLM outputs. After self-review, the second-annotator masks were frozen before quantitative analysis and were used only for the supplementary consistency analysis; they did not replace any of the 1500 primary reference masks. No adjudication procedure was performed.

Inter-annotator agreement within the 150-view subset was quantified using pixel-level intersection over union (IoU) and the Dice similarity coefficient (Dice) between the primary reference masks and the second-annotator masks. On the same subset, the unmodified initial Grounding DINO–SAM candidate masks, generated before Rule-weighted or Structured VLM processing, were also compared with the second-annotator masks using IoU and Dice to characterize upstream 2D mask quality. All 150 prespecified views were retained, and no view was excluded on the basis of mask quality or agreement. These supplementary analyses were descriptive, and no additional hypothesis tests were performed.

For each evaluation view, the cleaned mesh was projected onto the image plane to obtain the projected region S, which was compared with the corresponding manual reference mask R. IoU was used to quantify the overall overlap between the projected region and the manual reference mask. The leakage rate (Leak) was defined as the proportion of the projected region lying outside the manual reference mask and was used to reflect the risk of residual non-target regions, such as the background, bed surface, or supports. Coverage was defined as the proportion of the manual reference mask covered by the projection and was used to reflect the extent to which the target body surface region was retained.

Because manually annotated 2D boundaries, 3D projection, and rasterization may introduce small pixel-level boundary deviations, the 2-pixel tolerant leakage rate (TLeak_2_) was also calculated. Specifically, the manual reference mask R was dilated outward by 2 pixels at the original evaluation-image resolution to obtain $D_{2}(R)$. The proportion of the projected region S that remained outside this dilated region was then calculated. This metric reduces the influence of minor boundary misalignment on leakage estimation and places greater emphasis on background leakage extending beyond the boundary-tolerance range.

The balanced score (Balanced) was prespecified as the primary composite metric and was defined as the IoU minus the tolerant leakage rate. This metric jointly accounts for high contour overlap and substantive leakage beyond the tolerated boundary; a higher value indicates better reprojection quality in the evaluation view. The balanced score was used for the overall comparison, while IoU, tolerant leakage rate, and Coverage were reported as formal individual outcomes. The standard leakage rate was used to define the spillover proportion without boundary tolerance and served as an internal verification metric rather than a formally reported outcome. IoU, standard leakage rate, tolerant leakage rate, Coverage, and the balanced score were calculated using Equations (14)–(18), respectively.

(14) $$\mathrm{IoU}\left(S,R\right)=\frac{\left|S\cap R\right|}{\left|S\cup R\right|}$$

(15) $$\mathrm{Leak}\left(S,R\right)=\frac{\left|S\setminus R\right|}{\left|S\right|}$$

(16) $${\mathrm{TLeak}}_{2}\left(S,R\right)=\frac{\left|S\setminus D_{2}\left(R\right)\right|}{\left|S\right|}$$

(17) $$\mathrm{Coverage}\left(S,R\right)=\frac{\left|S\cap R\right|}{\left|R\right|}$$

(18) $$\mathrm{Balanced}\left(S,R\right)=\mathrm{IoU}\left(S,R\right)-{\mathrm{TLeak}}_{2}\left(S,R\right)$$

### 2.7. Implementation Details and Reproducibility

The structured VLM-based quality assessment used the API model identifier qwen3.7-plus provided by Alibaba Cloud Model Studio [36]. The available archived records do not retain an immutable provider-side snapshot identifier; model identity is therefore reported at the API-identifier level. For each voting view, the model inputs consisted of one mask-overlay image and one structured prompt. The sampling temperature was set to 0, the API timeout was set to 120 s, and the runtime configuration allowed up to two retries.

The archived quality-scoring dataset comprised 6000 voting-view records (150 acquisition–reconstruction trials × 40 views), with one valid JSONL record for each trial–view identifier. In 631 records (10.5%), the VLM response was not successfully parsed and the prespecified deterministic fallback based on mask statistics was applied; 627 carried the data_inspection_failed tag and four reflected API or model-call exceptions. runtime_fallback was a derived post-processing category encompassing these exception states rather than an additional raw-record label. After deterministic risk suppression, 613 fallback views received a final weight of 0.50 and 18 views with additional leakage evidence received 0.00. Because per-call exception messages, HTTP status codes, and retry states were not retained for the primary run, specific failure mechanisms were not assigned retrospectively. Raw responses, prompts, final weights, and reference-mask checksums were retained for verification. Acquisition-batch identifiers were unavailable; therefore, no batch-clustered resampling was performed.

To assess view-level Structured VLM repeatability, a prespecified stratified set of 300 inputs was constructed by selecting one representative and one challenge voting view from each of the 150 acquisition–reconstruction trials. The challenge stratum enriched historically fallback-prone inputs; the two strata were therefore analyzed separately, with pooled results treated as descriptive only. After the input identities, overlays, prompts, and order were frozen, each input underwent three fresh calls, yielding 900 logical calls in total. The calls used qwen3.7-plus, a temperature of 0, a 240 s timeout, at most two retries, and single-worker round-major execution. The 240 s timeout applied only to this experiment and did not alter the primary 6000-view analysis.

The primary repeatability analysis included only inputs with three successfully parsed, decision-complete, non-fallback records; a secondary end-to-end analysis retained valid deterministic fallback decisions. Outcomes included three-run exact agreement for final_action, the model-reported final_weight, the mapped weight, the final view_weight, and diagnostic and risk tags, together with Fleiss’ κ for final_action. Percentile 95% confidence intervals were estimated by trial-clustered bootstrap resampling with 5000 repetitions and a random seed of 14. This experiment assessed view-level decisions only and did not regenerate 2D masks, rerun 3D mesh voting, or reassess held-out reprojection performance. The archived files supporting the supplementary analyses are also provided in Supplementary File S1. All software components were used in the following versions: Python 3.10.20, PyTorch 2.7.1 (CUDA 11.8, cuDNN 9.1.0), OpenCV 4.13.0, NumPy 2.2.6, SciPy 1.15.3, scikit-learn 1.7.2, matplotlib 3.10.9 and Open3D 0.19.0; candidate 2D masks were generated with Grounding DINO 0.1.0 (Swin-T OGC checkpoint) and Segment Anything (SAM 1.0, ViT-B checkpoint), and mesh visualizations were rendered in Blender 5.1.0.

### 2.8. Statistical Analysis

All reprojection metrics were first calculated at the individual evaluation-view level. Within each acquisition–reconstruction trial, an equally weighted arithmetic mean was then calculated across the 10 evaluation views. Consequently, each method yielded 150 trial-level observations for each formally reported metric. The primary analysis used the 150 acquisition–reconstruction trials as the statistical units. The balanced score was prespecified as the primary outcome, and the tolerant leakage rate was prespecified as the secondary outcome. IoU and Coverage were reported as descriptive secondary outcomes.

Trial-level continuous outcomes for each method were summarized as the mean ± sample standard deviation (SD). The 95% confidence intervals (CIs) were estimated using the percentile-based nonparametric bootstrap method [37]. Grouped resampling was performed according to the acquisition–reconstruction trial identifier (ID), with each resample simultaneously retaining all three methods and all 10 evaluation views associated with the same trial. After 5000 repetitions, the 2.5th and 97.5th percentiles of the distribution of resampled means were used as the 95% CI limits, with the random seed fixed at 14. Paired tests were based on 150 trial-level paired differences. Comparisons between Structured VLM voting and Hard voting and between Structured VLM voting and Rule-weighted voting were performed separately for the primary balanced-score metric family and the prespecified secondary tolerant-leakage-rate metric family. Within each metric family, the two comparisons were conducted using two-sided Wilcoxon signed-rank tests, followed by separate Holm–Bonferroni correction [38]. An absolute paired difference of less than $1\times 10^{-12}$ was classified as a tie. This cutoff was used as a numerical-equivalence tolerance for residual round-off after division, averaging, and subtraction, rather than because double-precision floating-point arithmetic was considered inadequate. In the analyzed data, the smallest retained nonzero paired difference was $5.2\times 10^{-6}$, more than six orders of magnitude larger than the cutoff; treating only exact zeros as ties or using tolerances up to $1\times 10^{-9}$ yielded identical nonzero-pair counts, Wilcoxon statistics, and *p* values. The Wilcoxon tests included only nonzero trial-level differences exceeding this threshold.

To quantify practical significance, effect sizes were calculated from the same 150 trial-level paired differences used in each comparison. Let $d_{j}=x_{VLM,j}-x_{baseline,j}$ denote the paired difference for trial j. The paired standardized mean difference was calculated as Cohen’s $d_{z}=\frac{\bar{d}}{s_{d}}$, where $\bar{d}$ and $s_{d}$ are the mean and sample standard deviation of all 150 paired differences. Percentile bootstrap 95% confidence intervals for $d_{z}$ were obtained from 5000 trial-level resamples using a random seed of 14. The matched-pairs rank-biserial correlation was calculated as $r_{rb}=\frac{\left(R_{+}\;-\;R_{-}\right)}{\left(R_{+}\;+\;R_{-}\right)}$, where $R_{+}$ and $R_{-}$ denote the positive- and negative-rank sums after excluding ties using the same ${1\times 10}^{-12}$ threshold. Effect-size signs followed Structured VLM voting minus the corresponding baseline; therefore, positive values favored Structured VLM voting for the Balanced score, whereas negative values favored it for the tolerant leakage rate. Statistical significance was defined as a two-sided Holm-adjusted *p* < 0.05. To examine whether runtime fallback was associated with trial-level performance differences, the fallback rate for each trial was defined as the number of views classified as runtime_fallback divided by its 40 voting views. Balanced-score gain was defined as Structured VLM voting minus Rule-weighted voting, whereas tolerant-leakage-rate improvement was defined as Rule-weighted voting minus Structured VLM voting so that larger values favored the structured framework. Spearman’s rank correlation coefficient ($r_{s}$) was calculated between the fallback rate and each improvement measure across all 150 trials, with percentile-bootstrap 95% confidence intervals obtained from 5000 trial-level resamples using a random seed of 14 and Holm correction applied across the two correlations. An exploratory sensitivity analysis repeated these correlations in the 88 trials containing at least one fallback view. Supplementary sensitivity, annotation-consistency, fallback, and VLM-repeatability analyses were descriptive and were not included in the confirmatory testing families or used to select nominal parameter settings.

## 3. Results

### 3.1. Evaluation Data Completeness, Statistical Unit, and Supplementary 2D Mask Agreement

The manual reference-mask evaluation began with 150 separate acquisition–reconstruction trials. All 150 trials met the inclusion criteria, and no trial or evaluation view was excluded. Each trial contained 10 prespecified evaluation views held out from semantic cleanup, yielding a total of 1500 evaluation views. All three comparison methods produced valid outputs for all 150 trials. Mesh reprojection metrics were first calculated at the individual evaluation-view level and then averaged with equal weights across the 10 evaluation views within each trial. The formal statistical analyses were performed using the 150 trial-level results as the statistical units.

In the prespecified supplementary subset of 150 views, all views were retained. Agreement between the primary reference masks and the second-annotator masks was high, with an IoU of 0.9937 ± 0.0031 (95% CI [0.9932, 0.9942]) and a Dice coefficient of 0.9968 ± 0.0016 (95% CI [0.9966, 0.9971]). Against the same second-annotator masks, the unmodified initial Grounding DINO–SAM candidate masks achieved an IoU of 0.9935 ± 0.0031 (95% CI [0.9930, 0.9940]) and a Dice coefficient of 0.9968 ± 0.0016 (95% CI [0.9965, 0.9970]).

These complementary descriptive comparisons indicated high inter-annotator consistency and high agreement between the upstream 2D candidate masks and the second-annotator masks within the evaluated subset.

### 3.2. Overall Trial-Level Results of the Three Methods

**Figure 3:**
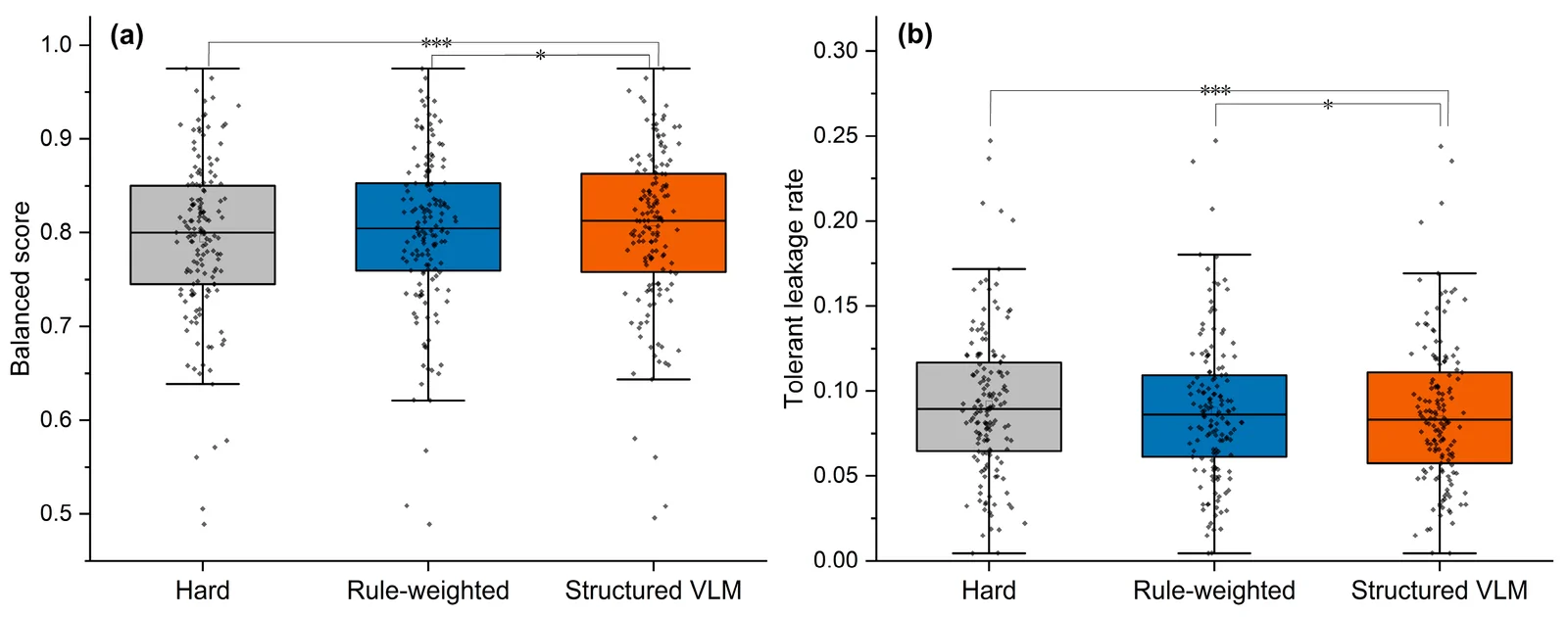
Trial-level distributions of (**a**) the Balanced score and (**b**) the tolerant leakage rate across the three methods. Each point represents the mean across 10 evaluation views from one of the 150 acquisition–reconstruction trials. Brackets indicate two-sided paired Wilcoxon signed-rank tests with Holm–Bonferroni correction; * p < 0.05, *** *p* < 0.001.

The trial-level results and paired comparisons for the three comparison methods are summarized in Table 5 (Parts A and B), and the trial-level distributions of the balanced score and tolerant leakage rate are shown in Figure 3. Table 5A reports the mean, SD, and 95% CI for each method, whereas Table 5B reports the trial-level paired differences between Structured VLM voting and the two baseline methods.

For the prespecified primary outcome, the balanced score, Structured VLM voting achieved a mean of 0.804, higher than 0.801 for Rule-weighted voting and 0.793 for Hard voting. Trial-level paired comparisons showed that the mean difference between Structured VLM voting and Hard voting was 0.0110 (95% CI [0.0080, 0.0140], *p* < 0.001), whereas the mean difference between Structured VLM voting and Rule-weighted voting was 0.0032 (95% CI [0.0004, 0.0062], *p* = 0.029). The corresponding effect sizes were Cohen’s $d_{z}=0.584$ (95% CI [0.499, 0.688]) and matched-pairs rank-biserial correlation $r_{rb}=0.950$ relative to Hard voting, and $d_{z}=0.175$ (95% CI [0.022, 0.327]) and $r_{rb}=0.279$ relative to Rule-weighted voting. The gain over Rule-weighted voting was therefore statistically significant but small in absolute magnitude.

For the prespecified secondary outcome, the tolerant leakage rate, Structured VLM voting had the lowest trial-level mean among the three methods (0.087), compared with 0.089 for Rule-weighted voting and 0.092 for Hard voting. Relative to Hard voting, Structured VLM voting yielded a mean difference of −0.0054 (95% CI [−0.0070, −0.0040], *p* < 0.001). Relative to Rule-weighted voting, the mean difference was −0.0016 (95% CI [−0.0030, −0.0002], *p* = 0.033). The corresponding effect sizes were $d_{z}=-0.581$ (95% CI [−0.685, −0.495]) and $r_{rb}=-0.949$ relative to Hard voting, and $d_{z}=-0.171$ (95% CI [−0.325, −0.019]) and $r_{rb}=-0.273$ relative to Rule-weighted voting. Both leakage-rate reductions remained statistically significant after Holm correction, although the absolute reduction relative to Rule-weighted voting was small. Across the three methods, mean Coverage ranged from 0.992 to 0.993, and mean IoU ranged from 0.886 to 0.891.

Figure 3 shows the distributions of the metrics reported in Table 5 across the 150 acquisition–reconstruction trials, illustrating the range of variability in the trial-level results for each method.

### 3.3. Paired Differences Relative to Rule-Weighted Voting

**Figure 4:**
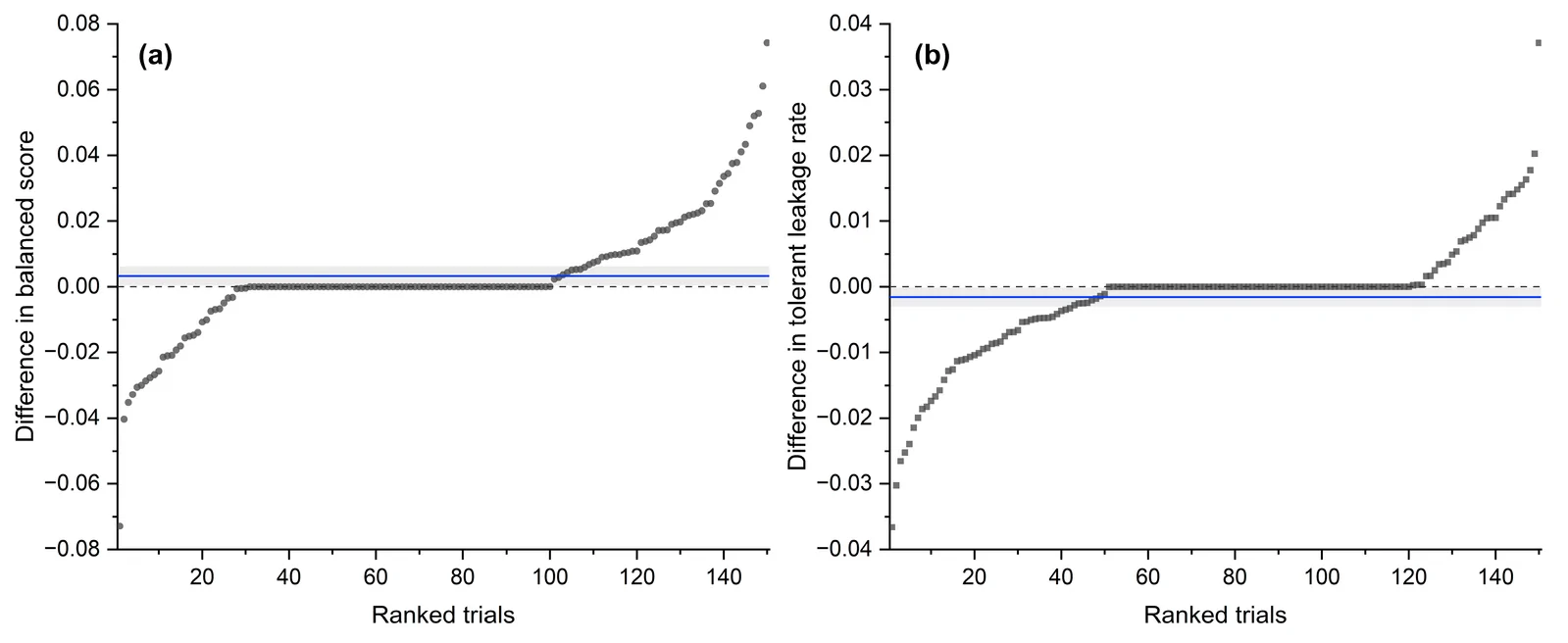
Ranked trial-level paired differences between Structured VLM voting and Rule-weighted voting for (**a**) the Balanced score and (**b**) the tolerant leakage rate. Differences are defined as Structured VLM minus Rule-weighted voting, and trials are independently ordered within each panel. The blue line and gray band denote the mean paired difference and its 95% bootstrap CI, respectively; the dashed line denotes zero. Positive values in (**a**) and negative values in (**b**) favor Structured VLM voting.

Figure 4 presents the trial-level paired differences between Structured VLM voting and Rule-weighted voting. The trials in the two panels were independently sorted in ascending order according to the difference in the corresponding metric. The horizontal axis indicates the trial rank within each panel; it does not represent the acquisition order or imply a one-to-one correspondence between trials across the two panels. The zero-reference line distinguishes relative improvement from relative deterioration.

For the balanced-score differences, 51/150 trials had positive values, 30/150 had negative values, and 69/150 were ties; after excluding ties, 81 nonzero paired differences remained. For the tolerant-leakage-rate differences, 51/150 trials had negative values, 30/150 had positive values, and 69/150 were ties, again yielding 81 nonzero paired differences after excluding ties. In the tolerant-leakage-rate panel, negative values indicate lower leakage with Structured VLM voting. The paired reduction remained statistically significant after Holm correction (*p* = 0.033).

### 3.4. Qualitative Comparison of 2D Reprojections and 3D Meshes

**Figure 5:**
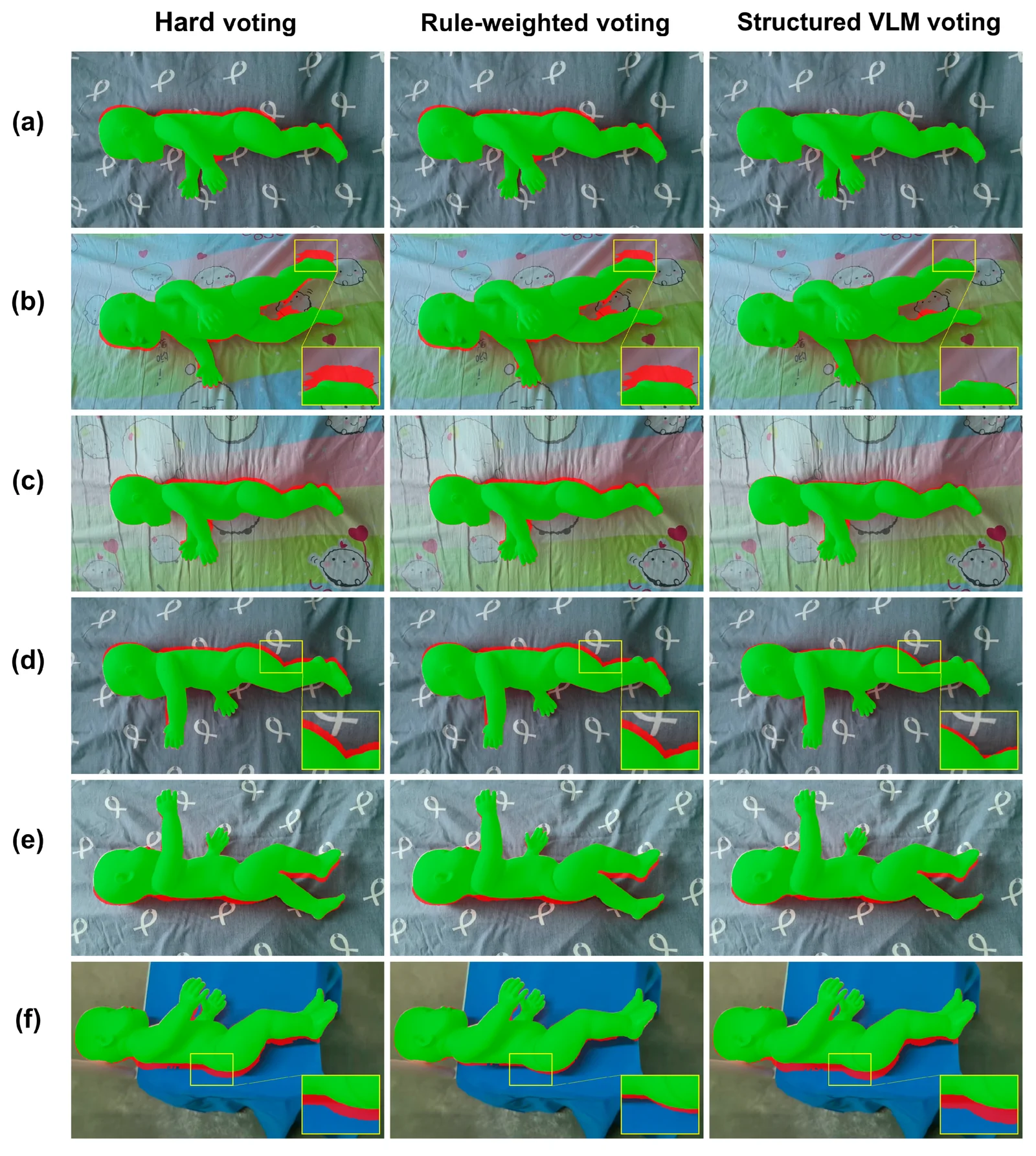
Two-dimensional reprojection overlays for Hard, Rule-weighted, and Structured VLM voting (columns) in six representative trials (rows (**a**–**f**)). Green denotes overlap with the manual reference mask and red denotes leakage; insets enlarge the boxed regions. Rows correspond to those in Figure 6.

**Figure 6:**
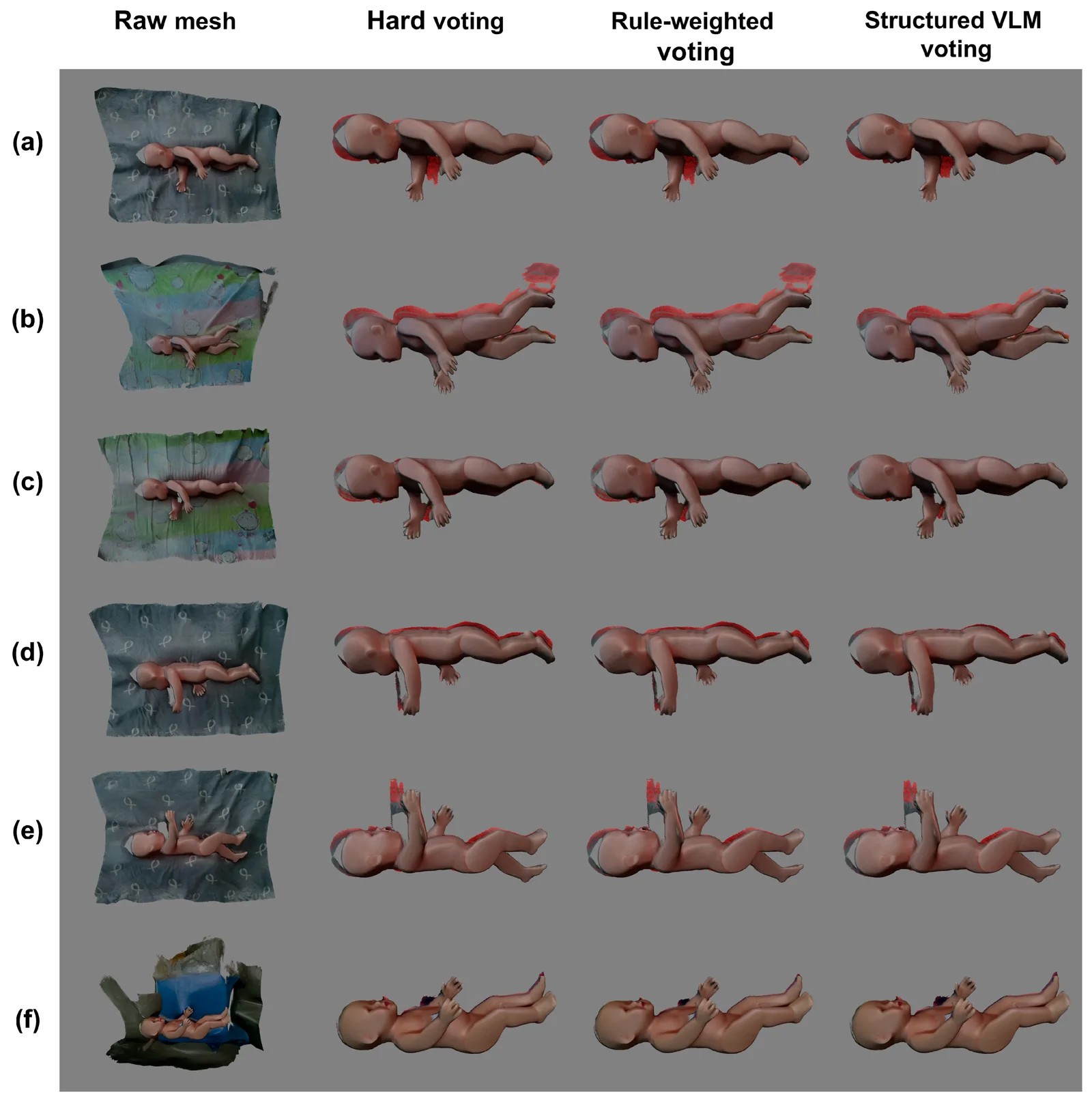
Three-dimensional mesh comparison for the six trials shown in Figure 5. Rows (**a**–**f**) correspond to the same six trials, in the same order; columns show the raw mesh, Hard voting, Rule-weighted voting, and Structured VLM voting; red highlights residual non-target geometry.

Figure 5 and Figure 6 illustrate qualitative phenomena that can be cross-referenced with the trial-level statistical results. To ensure row-wise correspondence between the qualitative 2D reprojection and 3D mesh visualizations, both figures use the same six prespecified representative examples. The first three rows show examples in which Structured VLM voting produced relatively clear improvements in the balanced score and leakage suppression. Row (d) presents a representative example in which Coverage was largely maintained while the tolerant leakage rate was reduced. Row (e) shows a representative example with a moderate improvement, whereas row (f) presents a boundary case in which Rule-weighted voting outperformed Structured VLM voting. These examples were selected solely to illustrate typical visual phenomena and methodological boundaries and were not used to estimate the frequency of these phenomena across all trials.

The visual differences in the 2D overlays were primarily concentrated in the red leakage regions. The first five examples suggest that Structured VLM voting can reduce some projection spillover in scenes involving different background cloths, bed surfaces, or nearby supports. Row (f) was retained as a boundary case, showing that Rule-weighted voting may still produce less visible leakage in the presence of a complex support background.

Figure 6 uses the same sequence of six acquisition–reconstruction trials as Figure 5 and maintains a consistent viewing pose, scale, rendering parameters, and cropping range across all columns. The 2D overlays depict the projected contour in an individual evaluation view, whereas the 3D mesh visualizations show the overall surface structure, residual background cloth or supports, and the integrity of the main body surface. The two visualization types therefore provide complementary qualitative information.

In the 3D mesh views, the raw meshes generally contained non-target structures such as background cloth, bed surfaces, or supports. All three cleanup methods preserved the overall body surface morphology, although they differed in residual components and local connectivity. The first five examples primarily illustrate the visual reduction in residual background and support structures, whereas row (f) shows that Rule-weighted voting produced a cleaner mesh in this boundary scenario. Figure 6 is intended only to provide a qualitative visualization of 3D surface morphology and residual non-target structures. The manually annotated 2D reference masks served solely as reference standards for 2D reprojection evaluation and are not equivalent to a 3D clinical gold standard.

### 3.5. Supplementary Analyses and Runtime Records

**Figure 7:**
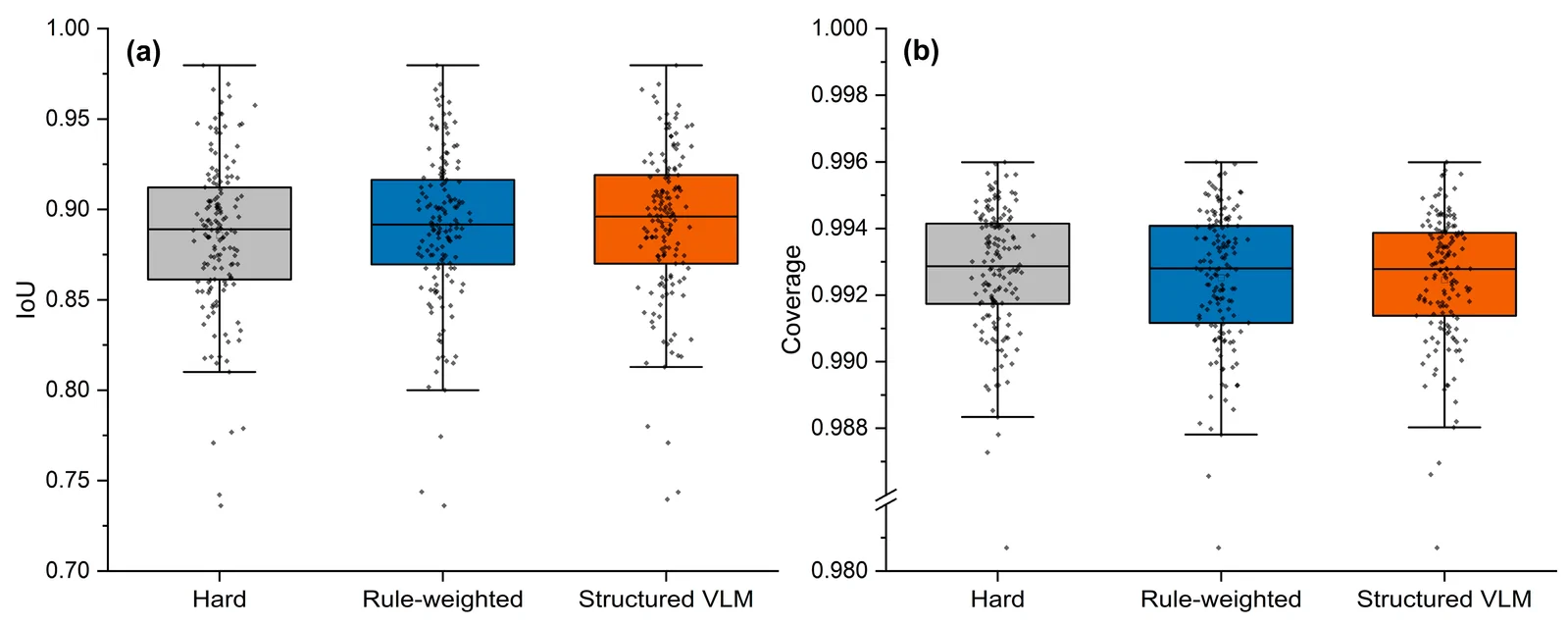
Trial-level distributions of (**a**) IoU and (**b**) Coverage across the three methods. Each point represents the mean across 10 evaluation views from one acquisition–reconstruction trial; the y-axis in panel (**b**) uses a broken scale.

Figure 7 further shows the trial-level distributions of IoU and Coverage. The mean IoU values were 0.886 for Hard voting, 0.890 for Rule-weighted voting, and 0.891 for Structured VLM voting. The corresponding mean Coverage values were 0.993, 0.993, and 0.992, respectively, with generally small differences among the methods.

**Figure 8:**
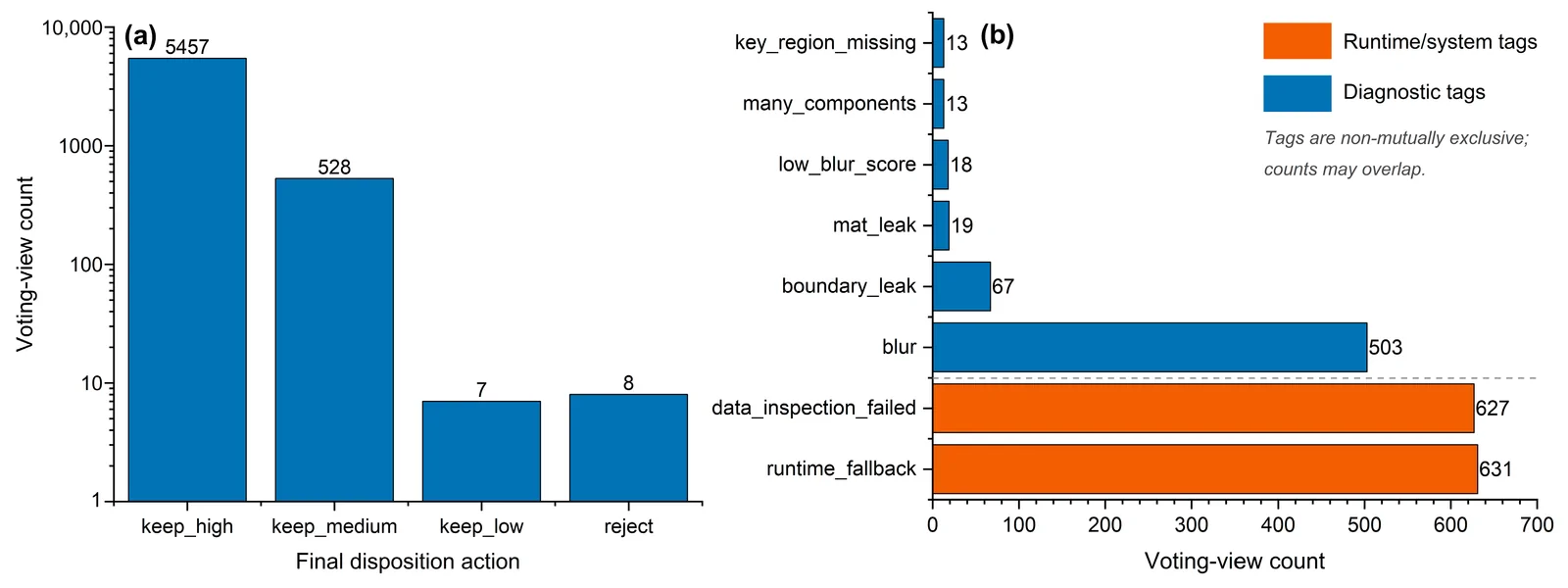
Summary of structured VLM view-quality assessment across 6000 voting views: (**a**) final disposition actions (final_action) after normalization and fallback processing, shown on a logarithmic scale; and (**b**) diagnostic tags (blue) and runtime/system labels or categories (orange). In panel (**b**), the dashed line separates these two tag groups. Counts in panel (**b**) are non-mutually exclusive.

Figure 8 and the runtime records summarize the distribution of final disposition actions after normalization and fallback processing, together with the runtime audit results. Among the 6000 voting-view records, 5457 (91.0%) were classified as high-quality retention, 528 (8.8%) as medium-quality retention, 7 (0.1%) as low-quality retention, and 8 (0.1%) as rejection. The runtime audit showed that 631 records (10.5%), distributed across 88 acquisition–reconstruction trials, did not yield successfully parsed VLM responses. Of these, 627 carried the data_inspection_failed tag, whereas the remaining 4 corresponded to API or model-call exceptions. Post-processing grouped all 631 records into the runtime_fallback statistical category. Notably, runtime_fallback is a higher-level fallback category generated during post-processing, whereas data_inspection_failed is a specific failure tag in the raw runtime records. The 627 records carrying the data_inspection_failed tag are included within the 631 records counted as runtime_fallback; therefore, their frequencies should not be summed directly. After deterministic risk suppression, 613 fallback views were assigned a final weight of 0.50, whereas 18 fallback views that also exhibited background-leakage evidence were suppressed to 0.00. The final disposition actions after normalization and fallback processing, runtime audit status, and final voting weights after deterministic risk suppression represent different levels of information and should not be treated as equivalent. No invalid JSON records were identified in the raw-response audit, and no duplicate trial–view pairs were found in the final scoring file.

At the trial level, the fallback rate was positively associated with both the Balanced-score gain and the tolerant-leakage-rate improvement of the complete Structured VLM framework relative to Rule-weighted voting (Spearman’s $r_{s}=0.608$, 95% CI [0.461, 0.733], and $r_{s}=0.607$, 95% CI [0.460, 0.733], respectively; Holm-adjusted *p* < 0.001 for both). In the 88 trials containing at least one fallback view, the corresponding correlations were $r_{s}=0.663$, and $r_{s}=0.661$. Because the Balanced score incorporates the tolerant leakage rate, the two improvement measures were nearly perfectly correlated and therefore represent two measures of essentially the same performance pattern. The observed association reflects the complete framework, including deterministic fallback handling and risk suppression, and therefore does not isolate a causal contribution from successful VLM reasoning.

### 3.6. Sensitivity Analyses

#### 3.6.1. Sensitivity to the Numbers of Voting and Evaluation Views

Within the common OpenCV 4.13.0 rasterization backend used for this descriptive analysis, changes in the number of voting views produced nonmonotonic group-level differences. With the fixed retention threshold T=4, mean Coverage increased only slightly as K increased from 10 to 40 (approximately 0.991–0.992), whereas the Balanced score and IoU were highest at K=10, decreased at K=20 and K=30, and partially recovered at K=40. The K=10 setting also yielded the lowest TLeak_2_ together with slightly lower Coverage, consistent with a more conservative retention pattern. Across the K=20, K=30, and K=40 configurations, the range of the mean Balanced score was less than 0.008 for each method. Hard voting yielded the lowest mean Balanced score throughout, whereas Structured VLM voting and Rule-weighted voting were close; Rule-weighted voting was slightly higher only at K=20.

The fixed and proportional retention-threshold schemes produced nearly identical group means, with a maximum absolute difference of 0.0013 in the mean Balanced score, although the largest single-trial difference reached 0.1155 for Structured VLM voting at K=10. Accordingly, K=40 was retained as the prespecified engineering compromise providing the fullest tested angular coverage, rather than being interpreted as a numerically optimized setting.

Evaluation-view subsampling was more stable when at least six of the ten held-out views were retained. For M=6 and M=8, all enumerated subsets preserved the M=10 group-level ordering of the three methods and the directions of both Structured VLM-versus-baseline comparisons. At M=4, these relationships were preserved for IoU, TLeak_2_, and the Balanced score, whereas the same ordering and comparison directions were retained in 198 of the 210 subsets for Coverage. At M=2, occasional changes were observed for TLeak_2_ and Coverage. Because absolute metric values and trial-level rankings still varied across subsets, M=6 was not treated as equivalent to M=10, and the prespecified M=10 configuration was retained for the primary analysis.

#### 3.6.2. Sensitivity to Threshold and Weight Settings

The one-factor-at-a-time analysis of the 15 alternative mask-quality threshold settings affected the three methods unequally. Hard voting was nearly invariant, with a maximum absolute change of 0.0010 in its mean Balanced score, and Structured VLM voting was also relatively stable, with an absolute change not exceeding 0.0024 across all settings. Rule-weighted voting was more sensitive: its mean Balanced-score difference from the nominal setting ranged from −0.0075 at a blur-score threshold of 10.0 to +0.0260 at a threshold of 60.0. Some alternative settings therefore changed the ordering of Rule-weighted voting and Structured VLM voting. Because these settings constituted a prespecified descriptive stress test rather than a tuning procedure, the observed changes were not used to revise the nominal thresholds.

Varying the vertex-retention threshold from T=2 to T=8 had negligible effects on the group-level metrics. At T=2, T=3, and T=5, the final meshes were identical to those obtained at T=4 in all 450 method–trial combinations (150 trials × three methods). For the stricter settings from T=6 to T=8, only 6–27 combinations differed from the T=4 results. Nevertheless, the corresponding changes in the group means of the Balanced score, TLeak_2_, IoU, and Coverage remained below $1\times 10^{-4}$, and no empty output meshes occurred. This limited sensitivity is consistent with the simultaneous foreground-majority requirement and subsequent largest-connected-component post-processing, which together reduced the number of vertices determined solely by T. Across all tested T values, Structured VLM voting, Rule-weighted voting, and Hard voting retained their descending order for the Balanced score and IoU; the numerical order was reversed for TLeak_2_, for which lower values indicate better performance. These results support robustness over the tested range but do not identify T=4 as an optimal value.

The power transformation of the view weights produced a gradual shift in the retention–suppression balance. As γ increased from 0.50 to 1.50, the mean Balanced score increased from 0.7960 to 0.8052 for Rule-weighted voting and from 0.7973 to 0.8085 for Structured VLM voting. Over the same range, the mean TLeak_2_ decreased, whereas Coverage changed by no more than 0.0002. Structured VLM voting retained a higher mean Balanced score and a lower mean TLeak_2_ than Rule-weighted voting at every tested γ, and both weighted methods remained above the Hard voting baseline in mean Balanced score. The nominal setting γ=1.00 was retained because it was prespecified, rather than because it maximized the observed metrics.

#### 3.6.3. Sensitivity to the Deterministic Risk Cap

Changing only the support-related cap $\rho_{support}$ from its nominal value of 0.25 to either 0.00 or 0.50 altered the final weights of 63 of the 6000 voting views, distributed across 27 of the 150 acquisition–reconstruction trials. The mean Balanced scores were 0.8024, 0.8044, and 0.8039 for $\rho_{support}$ = 0.00, 0.25, and 0.50, respectively. Relative to the nominal setting, the mean paired Balanced-score differences, defined as the alternative setting minus the nominal setting, were −0.0020 (95% CI [−0.0039, −0.0004]) at $\rho_{support}=0.00$ and −0.0004 (95% CI [−0.0008, −0.0001]) at $\rho_{support}=0.50$. The nominal setting also yielded the highest mean IoU and the lowest mean TLeak_2_, whereas mean Coverage differed by less than 0.00005 across the three settings.

Thus, both complete suppression at 0.00 and weaker suppression at 0.50 were associated with slightly lower mean Balanced scores than the nominal setting. The value 0.25 therefore provided an intermediate engineering limit that retained a restricted contribution from masks potentially containing usable infant-surface information while applying stronger suppression than the 0.50 cap used for less severe boundary-related uncertainty. These results provide empirical support for the prespecified value of 0.25 within the current dataset and tested alternatives; they do not establish it as a mathematically optimal value, and the cap was not reselected on the basis of this analysis.

### 3.7. Repeatability of Structured VLM Decisions

Among inputs meeting the primary eligibility criteria—successful completion and parsing of all three calls, complete decision fields, and no fallback processing—three-run exact agreement for final_action was 96.3% (95% CI [92.6%, 99.3%]; Fleiss’ κ=0.878; n=136) in the representative stratum and 85.9% (95% CI [76.6%, 93.8%]; κ=0.803; n=64) in the challenge stratum. Exact agreement for the final view weight was 93.4% and 79.7%, respectively. Thus, successfully completed calls showed high repeatability of the principal structured decision fields, with lower agreement for the deliberately difficult challenge inputs.

At the call level, the API and parsing success rates were each 90.7% (408/450) in the representative stratum and 43.1% (194/450) in the challenge stratum. In particular, 256 of the 450 challenge calls (56.9%) returned HTTP 400. The challenge-stratum agreement estimates therefore describe the subset for which all three calls completed successfully and should be considered together with the observed call-failure rate. Neither the raw response text nor the complete normalized JSON record was exactly identical across all three calls in the primary eligible subsets, indicating that repeatability applied to the key structured decision fields rather than to the verbatim model output. Because the challenge inputs were enriched for historically fallback-prone cases, the two strata were analyzed separately. This experiment assessed only the repeatability of view-level Structured VLM decisions and did not repeat 2D mask generation, 3D mesh voting, or held-out reprojection evaluation.

## 4. Discussion

This study evaluated structured VLM-based view weighting for multi-view segmentation and infant phantom mesh cleanup. Structured VLM voting achieved the highest mean Balanced score and the lowest mean TLeak_2_, while mean Coverage remained similar across the three methods (Table 5). The improvement therefore primarily reflected suppression of non-target geometry rather than expansion of the retained surface. Relative to Rule-weighted voting, the paired Balanced-score difference was 0.0032 (95% CI [0.0004, 0.0062]; Holm-adjusted *p* = 0.029), and the paired TLeak_2_ difference was −0.0016 (95% CI [−0.0030, −0.0002]; Holm-adjusted *p* = 0.033). Both differences were statistically significant, although their absolute magnitudes were small. Thus, the advantage over Hard voting was clearer, whereas the incremental gain over the stronger Rule-weighted baseline remained modest. These findings support structured VLM weighting as an upstream mesh-cleanup strategy, but they do not demonstrate improvements in ultrasound image quality, robotic trajectory execution, or probe–surface interaction.

Representative related work spans robotic-ultrasound planning and control [9,10,11,12,13,14,15], promptable and medical 2D segmentation [16,17,18,19,20], classical multi-view consistency and view selection [21,22], Gaussian scene reconstruction [23,24], 2D-to-3D semantic fusion and open-vocabulary 3D segmentation [25,26,27,28,29,30], and vision–language modeling [31,32,33,34,35]. Table 6 positions the proposed method relative to these categories. Because the cited studies use different datasets, task definitions, output representations, and evaluation metrics, and none evaluates infant-phantom surface-mesh cleanup for DDH robotic-ultrasound preprocessing, the comparison is qualitative rather than a cross-dataset numerical ranking.

Table 6 shows that the cited approaches address complementary stages of the present pipeline rather than directly interchangeable baselines. The proposed method is distinguished by structured assessment of candidate-mask reliability before deterministic target/non-target voting on an existing surface mesh.

In this study, the VLM neither directly generated the final segmentation nor modified the 3D mesh. Instead, it converted mask completeness, background-leakage risk, and retention of task-relevant regions into view weights, after which deterministic rules and vertex voting generated the cleaned mesh. The small but statistically significant Balanced-score gain and TLeak_2_ reduction over Rule-weighted voting suggest that structured assessment may add task-context information beyond handcrafted statistics; however, these results should not be interpreted as evidence of a general VLM advantage in mesh cleanup.

Body-surface geometry contributes to trajectory planning, scan-region definition, and interaction control in robotic ultrasound [9,10,11,12]. However, the absolute improvements over Rule-weighted voting were small (Balanced score, +0.0032; TLeak_2_, −0.0016), and the present study did not evaluate whether these improvements translate into better path feasibility, collision clearance, surface-normal accuracy, or probe–surface contact. Structured VLM assessment also introduces model-call, parsing, retry, and fallback-handling overhead. Because end-to-end latency and resource consumption were not benchmarked, whether the observed gain justifies the additional computational cost remains undetermined.

The qualitative examples were consistent with the trial-level findings while also showing a complex-support case in which Rule-weighted voting produced a cleaner result. Because these examples were selected for illustration, inference rests on the trial-level quantitative analysis.

Several limitations concern the data and reference standard. The 150 acquisition–reconstruction trials were separate technical repeats obtained from one physical infant phantom under controlled conditions, rather than observations from independent subjects. Variations in pose, background, supports, illumination, camera trajectory, and acquisition session therefore do not capture inter-individual differences in body size, skin appearance, motion, or occlusion. The full set of 1500 reference masks was generated by one primary annotator; however, a blinded independent audit of 150 images by a second annotator yielded pairwise IoUs of approximately 0.994. This subset audit does not provide a complete multi-annotator reference standard. Moreover, although the evaluation views were excluded from semantic voting, they originated from the same acquisition–reconstruction pipeline and do not constitute external validation. The 2D reprojection metrics cannot fully assess 3D topology, depth error, local curvature, surface-normal accuracy, or robotic reachability.

Projection did not include explicit z-buffer occlusion testing, back-face culling, or normal-angle filtering. Sensitivity analyses showed that the comparison with Hard voting was stable over the tested settings, whereas the ordering between Structured VLM and Rule-weighted voting changed at K=20 and under four mask-quality-threshold alternatives. Repeatability was high among successfully completed calls, but the challenge-stratum failure rate limits end-to-end repeatability claims. Robustness across alternative VLMs, prompts, segmentation models, and reconstruction methods remains to be established.

## 5. Conclusions

This study established a structured vision–language model (VLM)-based method for infant surface-mesh cleanup in robotic ultrasound preprocessing. Rather than allowing the VLM to generate the final segmentation or directly modify the mesh, the method uses structured VLM assessment to estimate candidate-mask reliability, converts the assessment into bounded view weights, and performs mesh cleanup through deterministic multi-view vertex voting. This design provides an auditable connection between semantic view-quality assessment and geometric evidence aggregation.

Evaluation across 150 separate acquisition–reconstruction trials using one commercial infant phantom showed that Structured VLM voting achieved the highest mean Balanced score and the lowest mean TLeak_2_ among the three evaluated methods while maintaining comparable body-surface coverage. Supplementary analyses characterized annotation consistency, parameter sensitivity, fallback behavior, and the repeatability of successfully completed VLM decisions, providing additional evidence on the technical behavior and reproducibility of the workflow under the tested conditions without treating the nominal configuration as numerically optimized. These findings constitute preliminary engineering evidence for upstream infant surface-mesh preprocessing rather than evidence of clinical diagnosis or robotic scanning performance. Validation using real-infant data, independent 3D reference standards, occlusion-aware projection, and closed-loop robotic execution is required before downstream or clinical application.

## Figures and Tables

**Table 1:** Dataset size and key parameters for 2D segmentation and 3D voting under the nominal configuration.

Module	Parameter	Setting
Data Scale	Physical object and experimental unit	Infant phantom, multi-condition acquisition; 150 separate acquisition–reconstruction trials
Raw Multi-View Data	Number of camera views per trial	99–155; median 121
View Partitioning	Voting views and evaluation views	40 voting views per trial; 10 evaluation views per trial
Text-Guided Segmentation	Text prompt, detection threshold, model, and mask merging	Text prompt: “the smooth skin of the baby doll.”; Grounding DINO Swin-T OGC; SAM ViT-B; detection box score threshold: 0.35
View Quality Statistics	Nominal rule-based thresholds	Foreground ratio lower bound: 0.05; upper bound: 0.65; max connected components: 8; largest component ratio lower bound: 0.95; blur threshold: 30.0
3D Voting	Vertex retention threshold and voting mode	Vertex foreground support threshold: 4.0; conservative voting mode; background counter-evidence not decayed
Post-processing	Connected components	Retain largest connected triangle mesh and remove unreferenced vertices

Note to Table 1: The quoted phrase is the verbatim prompt used throughout; it was not selected through prompt optimization.

**Table 2:** Mask-quality labels and view weights used in Rule-weighted voting.

Quality Level	Trigger Condition	Quality Label	View Weight
Critical Anomaly	The 2D mask is empty, including cases where Grounding DINO detects no valid candidate bounding box	empty_mask	0.00
Critical Anomaly	Foreground pixel ratio greater than 0.65	high_fg_ratio	0.00
Fragmentation Anomaly	Number of connected components greater than 8	many_components	0.50
Fragmentation Anomaly	Ratio of the largest connected component to foreground pixels less than 0.95	fragmented_mask	0.50
Minor Anomaly	Foreground pixel ratio less than 0.05	low_fg_ratio	0.70
Minor Anomaly	Laplacian blur score less than 30.0	low_blur_score	0.70
No Anomaly	None of the above anomaly rules triggered	ok	1.00

Note to Table 2: The weights are prespecified ordinal engineering values rather than calibrated probabilities. Values of 1.00, 0.70, 0.50, and 0.00 denote full foreground support, mild attenuation, half foreground support, and exclusion from voting, respectively. Sensitivity to their numerical scaling was evaluated using the power transformation in Equation (11), with the corresponding results reported in Section 3.6.2.

**Table 3:** Inputs, outputs, and verification objectives of the modules and roles in the structured VLM.

Module/Role	Actual Input	Structured Output	Verification Target or Weight Mapping
View-quality package	Raw image path, candidate mask, overlay, mask statistics, rule-based quality label, and prompt path	Single-view quality information package containing view identifier, evidence associations, quality statistics, and auxiliary tags	Raw image and mask used for construction and tracing; VLM API input: overlay and structured prompt only
Mask Quality Agent	Same overlay and same prompt	action, weight, tags, reason	Evaluates mask completeness, continuity, fragmentation, sharpness, and missing body surface areas
Background Leakage Agent	Same overlay and same prompt	action, weight, tags, reason	Evaluates leakage from supports, padding, cloth, table surface, and other non-infant regions
Task Region Agent	Same overlay and same prompt	action, weight, tags, reason	Evaluates whether DDH-relevant body surface regions such as the hip, buttock, abdomen, and thigh are retained
Judge Agent	Same input plus structured opinions from the three preceding roles	conflict_level, final_action, final_weight, final_tags, reflection, reason	Aggregates role opinions; applies conservative rules to determine final disposition in case of conflict
Disposition–Weight Mapping	keep_high, keep_medium, keep_low, and reject	1.00, 0.85, 0.50, and 0.00	final_action is mapped to the initial VLM weight; final_weight is used only for interpretation and auditing; and 3D voting reads the final view_weight generated through post-processing.

**Table 4:** Differences among the three comparison methods within the unified vertex-voting framework.

Method	View Weight Source	Foreground Support	Background-Opposition Accumulation	Primary Objective
Hard Voting	Equal weight for all views participating in voting	Unweighted accumulation	Unweighted accumulation	Serves as the baseline for multi-view semantic voting
Rule-weighted Voting	2D mask statistical rules	Weighted accumulation by rule-based weights	Background counter-evidence not attenuated	Reduces the influence of evidently low-quality masks on foreground retention
Structured VLM Voting	Structured VLM quality assessment and deterministic risk suppression	Weighted accumulation by final view weights	Background counter-evidence not attenuated	Further suppresses background and support-structure leakage risk in task context

**Table 5:** Trial-level reprojection results and paired comparisons of the three methods using manual reference-mask evaluation. Part (**A**): Trial-level metric means ± SD for the three methods, with 95% CIs shown in parentheses. Part (**B**): Trial-level paired differences between Structured VLM voting and the baseline methods. The mean difference was defined as Structured VLM voting minus the corresponding baseline. The balanced score was the prespecified primary outcome, and the tolerant leakage rate was the prespecified secondary outcome.

(**A**)					
**Method**	**Trials**	**Balanced Score**	**Tolerant Leakage Rate**	**IoU**	**Coverage**
Hard Voting	150	0.793 ± 0.086 (0.779–0.807)	0.092 ± 0.043 (0.085–0.099)	0.886 ± 0.043 (0.879–0.893)	0.993 ± 0.002 (0.992–0.993)
Rule-weighted Voting	150	0.801 ± 0.084 (0.787–0.815)	0.089 ± 0.042 (0.082–0.095)	0.890 ± 0.042 (0.883–0.896)	0.993 ± 0.002 (0.992–0.993)
Structured VLM Voting	150	0.804 ± 0.085 (0.790–0.818)	0.087 ± 0.043 (0.080–0.094)	0.891 ± 0.042 (0.884–0.898)	0.992 ± 0.002 (0.992–0.993)
(**B**)					
**Metric**	**Pairwise Comparison**		**Mean Difference**	**95% CI**	**Holm-Corrected *p*-Value**
Balanced Score	Structured VLM Voting minus Hard Voting		0.0110	[0.0080, 0.0140]	*p* < 0.001
Balanced Score	Structured VLM Voting minus Rule-weighted Voting		0.0032	[0.0004, 0.0062]	*p* = 0.029
Tolerant Leakage Rate	Structured VLM Voting minus Hard Voting		−0.0054	[−0.0070, −0.0040]	*p* < 0.001
Tolerant Leakage Rate	Structured VLM Voting minus Rule-weighted Voting		−0.0016	[−0.0030, −0.0002]	*p* = 0.033

Note to Table 5A: The 95% CIs are nonparametric bootstrap intervals for the trial-level mean of each method. Note to Table 5B: The 95% CIs are nonparametric bootstrap intervals for the trial-level mean paired differences. Holm-adjusted *p* values were obtained from two-sided Wilcoxon signed-rank tests. The balanced score and tolerant leakage rate were treated as separate comparison families, with the two baseline comparisons corrected within each family using the Holm–Bonferroni procedure.

**Table 6:** Task-level qualitative positioning of the proposed method relative to representative related approaches.

Category	Representative Works	Primary Task or Output	Treatment of View/Mask Reliability	Relation to the Present Study
Robotic-ultrasound systems	RobUSt, trajectory generation, compliant control, path planning, and automated scanning [9,10,11,12,13,14,15]	Robotic planning, control, acquisition, or reconstruction	Body-surface geometry is used as downstream input; semantic mask reliability is not the main target	Establishes the downstream application requirement
Promptable and medical 2D segmentation	SAM, Grounding DINO, MedSAM, MedCLIP-SAM, and AutoSAMUS [16,17,18,19,20]	2D object localization or mask generation	Primarily operates on individual images; no structured cross-view mask-quality weighting	Provides candidate 2D semantic evidence
Classical multi-view geometry	RANSAC and pixelwise view selection [21,22]	Robust model fitting and source-view selection	Uses geometric or photometric consistency	Provides complementary geometric reliability mechanisms
Gaussian scene reconstruction	3DGS and 2DGS [23,24]	Radiance-field rendering, geometric reconstruction, and mesh extraction	Optimizes scene primitives rather than semantic mask quality	Provides the upstream reconstructed representation
3D semantic fusion and segmentation	LangSplat, Gaussian Grouping, SA3D, OpenMask3D, Open3DIS, and Segment Any 3D Gaussians [25,26,27,28,29,30]	Scene querying, grouping, or 3D/open-vocabulary segmentation	Aggregates image features or 2D masks but is not organized around structured VLM assessment of each candidate mask	Closely related 2D-to-3D semantic transfer, but with different tasks and outputs
General and medical VLMs	VividMed, MLeVLM, CLIP, Qwen-VL, and biomedical Qwen2-VL [31,32,33,34,35]	Visual grounding, image–text alignment, or medical visual reasoning	Provides semantic reasoning but does not produce mesh-voting decisions or cleaned meshes	Motivates the structured view-quality assessor
Proposed method	Structured VLM voting	Binary target/non-target cleanup of an existing infant-phantom surface mesh	Structured mask assessment is converted into explicit view weights followed by deterministic risk suppression	Directly addresses the task studied here

## Data Availability

The processed data supporting the supplementary analyses are provided in Supplementary File S1. Additional study data are available from the corresponding author upon reasonable request.

## Supplementary Materials

The following supporting information can be downloaded at https://www.mdpi.com/article/10.3390/bioengineering13101213/s1: Supplementary File S1: Complete structured VLM prompt, deterministic decision rules, and archived files supporting the supplementary analyses.

## Abbreviations

The following abbreviations are used in this manuscript:

DDH	Developmental Dysplasia of the Hip
IoU	Intersection over Union
SAM	Segment Anything Model
TLeak_2_	2-pixel Tolerant Leakage Rate
VLM	Vision–Language Model
2DGS	2D Gaussian Splatting
